# Influenza Vaccination for Immunocompromised Patients: Systematic Review and Meta-Analysis from a Public Health Policy Perspective

**DOI:** 10.1371/journal.pone.0029249

**Published:** 2011-12-22

**Authors:** Charles R. Beck, Bruce C. McKenzie, Ahmed B. Hashim, Rebecca C. Harris, Arina Zanuzdana, Gabriel Agboado, Elizabeth Orton, Laura Béchard-Evans, Gemma Morgan, Charlotte Stevenson, Rachel Weston, Mitsuru Mukaigawara, Joanne Enstone, Glenda Augustine, Mobasher Butt, Sophie Kim, Richard Puleston, Girija Dabke, Robert Howard, Julie O'Boyle, Mary O'Brien, Lauren Ahyow, Helene Denness, Siobhan Farmer, Jose Figureroa, Paul Fisher, Felix Greaves, Munib Haroon, Sophie Haroon, Caroline Hird, Rachel Isba, David A. Ishola, Marko Kerac, Vivienne Parish, Jonathan Roberts, Julia Rosser, Sarah Theaker, Dean Wallace, Neil Wigglesworth, Liz Lingard, Yana Vinogradova, Hiroshi Horiuchi, Javier Peñalver, Jonathan S. Nguyen-Van-Tam

**Affiliations:** 1 Division of Epidemiology and Public Health, University of Nottingham, Nottingham, United Kingdom; 2 Global Influenza Programme, World Health Organization, Geneva, Switzerland; 3 Department of Public Health Medicine, University of Bielefeld, Bielefeld, Germany; 4 Public Health Directorate, National Health Services Blackpool, Blackpool, United Kingdom; 5 Division of Primary Care, University of Nottingham, Nottingham, United Kingdom; 6 Freelance, World Health Organization, Geneva, Switzerland; 7 South West (North) Health Protection Unit, Health Protection Agency, Gloucester, United Kingdom; 8 Cumbria and Lancashire Health Protection Unit, Health Protection Agency, Chorley, United Kingdom; 9 Health Protection Agency Yorkshire and Humber, Health Protection Agency, Leeds, United Kingdom; 10 Public Health Directorate, National Health Services Leicestershire County, Leicester, United Kingdom; 11 Department of Medicine, Brighton and Sussex University Hospital National Health Services Trust, Brighton, United Kingdom; 12 Law School, Harvard University, Cambridge, Massachusetts, United States of America; 13 Hampshire and Isle Of Wight Health Protection Unit, Health Protection Agency, Fareham, United Kingdom; 14 Public Health Directorate, National Health Services, Nottingham City, Nottingham, United Kingdom; 15 Public Health, Wessex Deanery, Winchester, United Kingdom; 16 Public Health Directorate, National Health Services Derbyshire County, Chesterfield, United Kingdom; 17 Public Health, Mersey Deanery, Liverpool, United Kingdom; 18 Medical School, Harvard University, Boston, Massachusetts, United States of America; 19 Public Health Directorate, Solihull National Health Services Primary Care Trust, Solihull, United Kingdom; 20 School of Public Health, Imperial College London, London, United Kingdom; 21 Childrens Services, Leicester Partnership Trust, Leicester, United Kingdom; 22 Health Protection Agency East Midlands, Health Protection Agency, Nottingham, United Kingdom; 23 Medical School, Lancaster University, Lancaster, United Kingdom; 24 Department of Infection and Population Health, University College London, London, United Kingdom; 25 Centre for International Health and Development, University College London, London, United Kingdom; 26 Public Health Directorate, National Health Services Northamptonshire, Northampton, United Kingdom; 27 Public Health Directorate, National Health Services Halton and St. Helens, Widnes, United Kingdom; 28 Public Health Directorate, NHS Nottinghamshire County, Mansfield, United Kingdom; 29 Public Health Directorate, NHS East Lancashire, Nelson, United Kingdom; 30 Health Protection Agency North East, Health Protection Agency, Newcastle upon Tyne, United Kingdom; 31 Faculty of Medicine, Tokyo Medical and Dental University, Tokyo, Japan; University of Hong Kong, Hong Kong

## Abstract

**Background:**

Immunocompromised patients are vulnerable to severe or complicated influenza infection. Vaccination is widely recommended for this group. This systematic review and meta-analysis assesses influenza vaccination for immunocompromised patients in terms of preventing influenza-like illness and laboratory confirmed influenza, serological response and adverse events.

**Methodology/Principal Findings:**

Electronic databases and grey literature were searched and records were screened against eligibility criteria. Data extraction and risk of bias assessments were performed in duplicate. Results were synthesised narratively and meta-analyses were conducted where feasible. Heterogeneity was assessed using I^2^ and publication bias was assessed using Begg's funnel plot and Egger's regression test. Many of the 209 eligible studies included an unclear or high risk of bias. Meta-analyses showed a significant effect of preventing influenza-like illness (odds ratio [OR] = 0.23; 95% confidence interval [CI] = 0.16–0.34; p<0.001) and laboratory confirmed influenza infection (OR = 0.15; 95% CI = 0.03–0.63; p = 0.01) through vaccinating immunocompromised patie nts compared to placebo or unvaccinated controls. We found no difference in the odds of influenza-like illness compared to vaccinated immunocompetent controls. The pooled odds of seroconversion were lower in vaccinated patients compared to immunocompetent controls for seasonal influenza A(H1N1), A(H3N2) and B. A similar trend was identified for seroprotection. Meta-analyses of seroconversion showed higher odds in vaccinated patients compared to placebo or unvaccinated controls, although this reached significance for influenza B only. Publication bias was not detected and narrative synthesis supported our findings. No consistent evidence of safety concerns was identified.

**Conclusions/Significance:**

Infection prevention and control strategies should recommend vaccinating immunocompromised patients. Potential for bias and confounding and the presence of heterogeneity mean the evidence reviewed is generally weak, although the directions of effects are consistent. Areas for further research are identified.

## Introduction

Respiratory disease is a leading cause of global mortality to which seasonal and pandemic influenza both make substantial contributions. For example, in the USA an estimated average 225,000 hospitalisations and 36,000 deaths per annum are attributable to seasonal influenza [1], [2]. Even the ‘mild’ 2009 influenza A(H1N1) pandemic was associated with substantial years of life lost due to mortality in younger age groups [3].

Patients with sub-optimal immune function due to disease or therapy (the immunocompromised) are recognised to be at increased risk from influenza-related complications, and are recommended for annual vaccination in many national vaccination guidelines. Concerns about influenza within immunocompromised populations include an impaired respo nse to vaccination and higher risk of complicated infection with increased mortality [4], greater and prolonged virus shedding with implications for control of transmission [5]–[8], the emergence of resistance to antiviral agents [9] and possible adverse effects of vaccination. The balance between potential benefit and harm resulting from vaccinating these groups has been hard to establish, with previous reviews finding few studies offering incontrovertible evidence of clinical protection [10]–[13]. There is uncertainty around thresholds for defining immunocompromise and the exte nt to which underlying aetiologies vary in their susceptibility to influenza and potentially their response to vaccine, with deference to clinical opinion in many cases [14]. A high burden of illness was recognised in immunocompromised patients during the 2009 influenza A(H1N1) pandemic, along with substantial nosocomial disease, proclaiming the need to re-visit the evidence base for influenza vaccination in these patients [8], [15]–[21].

We conducted a systematic review and meta-analysis to assess influenza vaccination for immunocompromised patients. We report the primary analysis and its interpretation from a public health policy perspective, to assess the overall evidence. A second manuscript will be submitted for publication which reports a secondary analysis of our data, stratified by aetiology of immunocompromise.

## Methods

An abbreviated study protocol is available from the National Institute for Health Research international prospective register of systematic reviews (PROSPERO) [22], and the full protocol and PRISMA checklist are available as supporting information (see Protocol S1 and Checklist S1). Minor amendments to the original protocol were conducted to clarify the search strategy and eligibility criteria.

The study population of interest comprised all persons immunocompromised due to primary immunodeficiency (genetic defects) or secondary immunodeficiency (such as HIV infection, malignancy, or receipt of immunosuppressive drugs). Immunocompromised populations were derived from World Health Organization (WHO) and United Kingdom (UK) Department of Health immunisation policy to prevent influenza infection [14], [23]. We additionally included malnutrition and tuberculosis as conditions commonly associated with immunocompromise in developing countries. Interventions of interest comprised vaccination against seasonal influenza or 2009 influenza A(H1N1) pandemic; restricted to experimental designs for seasonal influenza but with no limitation for pandemic studies where experimental approaches would have been ethically unfeasible in most circumstances. Comparative groups included vaccinated immunocompetent controls (VICT) and immunocompromised patients given placebo or no vaccination (PNV). Outcome measures corresponded to four research questions relevant to this review: prevention of clinically diagnosed influenza or influenza-like illness (ILI) and laboratory confirmed influenza infection, serological response, and adverse events associated with vaccination. Criteria for inclusion and exclusion of studies, established in advance of executing the search str ategy, are presented in Table 1 and information sources searched to identify relevant literature are shown in Table 2.

**Table 1:** Study eligibility criteria.

*Inclusion criteria*
Experimental studies or systematic reviews (± meta-analyses) reporting data on the efficacy, effectiveness, immunological response or adverse effects associated with influenza vaccination of immunocompromised patients to prevent infection from seasonal influenza or 2009 influenza A(H1N1) pandemic strain
Observational studies published during 2009 and 2010 reporting data on the efficacy, effectiveness, immunological response or adverse effects associated with influenza vaccination of immunocompromised patients to prevent infection from 2009 influenza A(H1N1) pandemic strain
Studies which recruited individuals of any age from any setting who are immunocompromised whether due to primary immunodeficiency (genetic defects) or secondary immunodeficiency (such as HIV infection, malignancy, poor nutritional status or use of immunosuppressive drugs)
No restriction is placed on the influenza vaccination dose, preparation, trade name, schedule or method of administration
Studies which report data from control or comparator treatments may include no vaccination, placebo vaccination or sham vaccination
Studies which have recruited immunocompromised patients and compare outcome measures with immunocompetent control study subjects
Studies which report data on at least one of the following outcome measures: rate of clinically diagnosed influenza or ILI/ITT patients, rate of laboratory confirmed influenza or ITTI patients, immunological response to vaccination, and adverse effects associated with vaccination
Full text manuscripts of studies which are publis hed in English, French, Spanish, Portuguese, Russian, or Japanese

*Exclusion criteria*
Any literature or search hit which does not describe outcome measures obtained from an experimental study, observational study, or systematic review (± meta-analysis)
Any systematic review (± meta-analysis) which has been superseded by an updated evidence synthesis (such as updated reviews published by the Cochrane Library)
Studies which report outcome measures associated with vaccination against avian influenza
Studies which do not report follow-up data of patients within 12 months of intervention
Studies which have recruited less than 5 subjects to the interve ntion arm or exposed group
Studies which have not recruited immunocompromised patients which include those aetiologies described in the protocol
Studies which compare vaccination with an active comparator and which do not report data from a control group of study subjects
Studies which compare vaccination only by route of administration or dosing schedules
Studies which report data from patients with drug induced immunosuppression where less than 80% of the study group are receiving immunosuppressive treatment*

*Applied to respiratory and autoimmune conditions only; no specification of dosage or duration of therapy.

**Table 2:** Information sources.

*Category*	*Source*
Databases	MEDLINE; EMBASE; CINAHL; Cochrane Central Register of Controlled Trials (CENTRAL); PubMed; WHO Regional Indexes; J-STAGE (Japanese language); Banque de Données en Santé Publique (BDSP, French language); Index-F (Spanish language); eLIBRARY (Russian language)
Evidence-based reviews	Bandolier; Cochrane Library: Database of Systematic Reviews (CDSR), Database of Abstracts of Reviews of Effects (DARE), National Health Service Health Technology Assessment (NHS HTA)
Guidelines	NHS Evidence: NHS Clinical Knowledge Summaries, National Library of Guidelines
Grey literature	Web of Science; NHS Evidence; OpenSIGLE; influenza vaccine manufacturers: GlaxoSmithKline, Novaratis, Sanofi Pasteur MSD, Abbott, CSL Limited, Medimmune, Crucell, Baxter; European Vaccine Manufacturers (Brussels); International Federation of Pharmaceutical Manufacturers Associations (Geneva/Zurich); consultation with domain expert (Bram Palache, Abbott)
Hand searching of journals	*Vaccine*
Reference tracking	Reference lists of all included studies
Citation tracking	Web of Science (Science Citation Index); Google Scholar
Internet searching	www.google.com; www.dh.gov.uk; www.hpa.org.uk; www.who.int; www.cdc.gov; www.flu.gov

### Search strategy and study selection

Single reviewers conducted searches during January 2011, based on the term construct used for MEDLINE (see Table S1), which was subsequently adapted or translated for other information sources as appropriate. No date limit for publication was applied to studies of seasonal influenza whilst a limit of 2009–10 was applied to studies pertaining to the 2009 influenza A(H1N1) pandemic. Results were limited to human subjects and language of publication restricted to English, French, Japanese, Portuguese, Spanish and Russian.

After removal of duplicates a three-stage screening process applied the eligibility criteria to all records. Screening at title, abstract and full text was managed primarily within EndNote® ×4.0.2 (Thomson Reuters, California, USA). Records in non-compatible formats or non-English languages were manually screened. Screening was un dertaken by two reviewers in parallel, with consensus by discussion and provision for arbitration by a third reviewer. Due to insufficient resources, Spanish and Portuguese literature was screened by one reviewer.

### Data collection

Data were extracted by two reviewers in parallel using a piloted template, with consensus by discussion and provision for arbitration by a third reviewer. No further data were sought from corresponding authors of eligible studies. Items extracted for study characteristics comprised country setting, objectives, design, sample size, methods of recruitment, inclusion and exclusion criteria, sequence generation, allocation, confounders and funding source. Population items comprised description of study groups, setting and stability of setting, age, sex, socioeconomic characteristics and risk factors for exposure to influenza. Intervention items comprised healthcare provider, setting in which health care delivered, description of intervention or exposure, vaccination type, route of administration, dosing schedule, and number of subjects allocated to and receiving the intervention or exposure. Outcome items comprised definition, measurement tool, timing and unit of measurements, blinding of assessors, duration of follow-up, number of measurements made (including withdrawals, exclusions and losses to follow-up), intervention and comparator results, detail of statistical analyses performed, and control for selection bias and confounding.

Extracted outcome data on immune response were classified according to Committee for Human Medicinal Products (CHMP) seroconversion and seroprotection criteria for each influenza subtype patients were vaccinated against [24]. Studies were excluded from meta-analysis if they did not provide data assessable against CHMP criteria or did not draw blood for serology at any time within 2–4 weeks post-vaccination. Geometric mean titre (GMT) and mean fold increase of haemagglutination inhibition (HI) levels pre- and post-vaccination were extracted. Adverse event data on local and systemic events were extracted according to CHMP criteria [24]. In addition, data on serious adverse events [25] and disease progression or clinical impact of immunocompromising condition were also extracted.

### Risk of bias in individual studies

Risk of bias was assessed at both study and outcome level using tools produced by the Cochrane Collaboration [26] for experimental and prospective cohort designs, Downs and Black [27] for observational designs (excluding prospective cohort studies) and the US Agency for Healthcare Research Quality (AHRQ) [28] for systematic reviews. Assessments were undertaken in parallel by two reviewers reaching consensus by discussion, with provision for arbitration by a third reviewer. Abstract-only records were not subject to assessment of risk of bias due to paucity of information. Domain-based risk of bias was used to inform narrative synthesis, thus avoiding overall scores in accordance with recommendations [26], [29].

### Summary measures

Descriptive statistics were calculated using Microsoft® Office Excel® 2007 version 12 (Microsoft Corporation, Richmond, USA). Where feasible, odds ratios including 95% confidence intervals and the standard error of the natural log odds ratio were calculated for input into meta-analyses.

### Synthesis of results

Primary analysis was designed to synthesize appraisal of methodological quality and extracted study data by means of tabulation, narrative and meta-analysis (where appropriate). With the exception of serological outcome measures, data pertaining to the 2009 influenza A(H1N1) pandemic were pooled together with seasonal influenza data in accordance with the research aim to assess overall evidence. Meta-analysis of pooled odds ratios estimated the effect size of vaccinating immunocompromised patients versus immunocompetent controls (VICT), and of immunocompromised patients receiving vaccination versus those receiving placebo or no vaccination (PNV). Meta-analyses were conducted using Stata® version 10 (StatCorp LP, Texas, USA) initially using a random effects model. Analyses were re-executed using a fixed effects model where heterogeneity was low (I^2^<40%) and abandoned where heterogeneity was high (I^2^>85%). Statistical significanc e of pooled odds was assumed at the 5% level and assessed using the *Χ* ^2^ test. Risk of publication bias for studies subject to meta-analysis was assessed visually using Begg's funnel plots and quantified using Egger's regression test. Sub-analysis sought to utilise the UN inequality-adjusted Human Development Index 2010 (UN HDI) [30] for stratification of countries by quartile of human development to assess the strength of evidence in low resource environments.

## Results

### Study selection and characteristics

**Figure 1:**
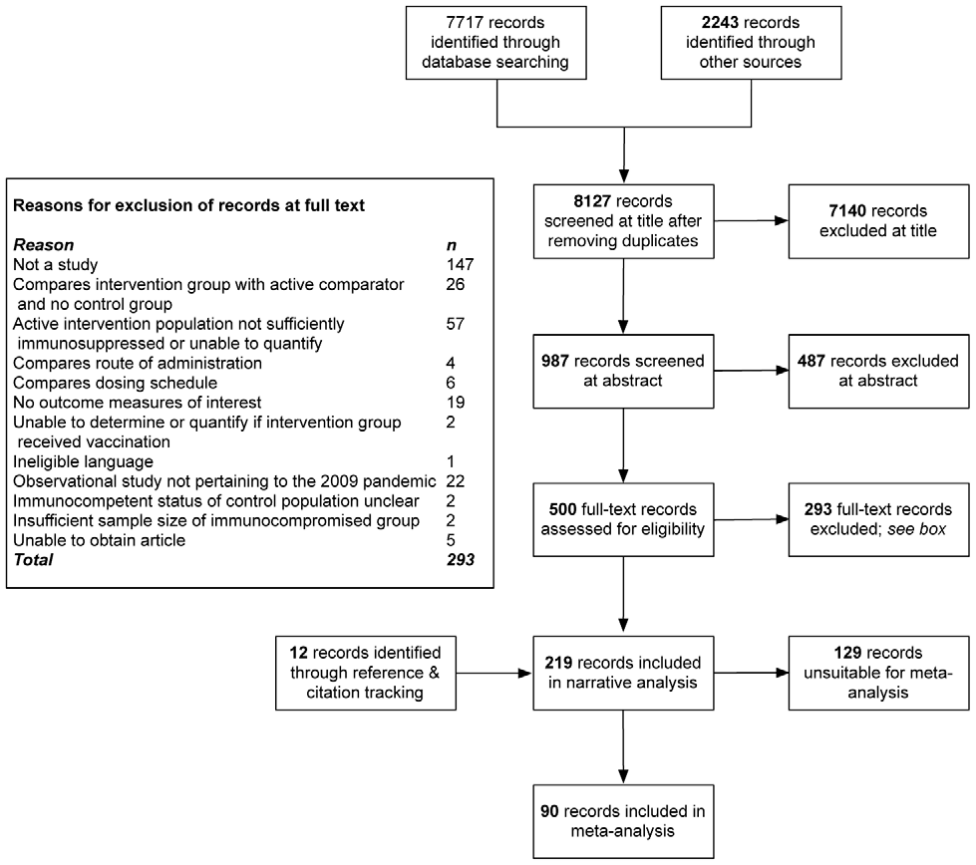
Summary of study selection process.

Figure 1 provides an account of the study selection process in the form of a PRISMA flow diagram [29]. The search strategy initially yielded 9,960 records (of which 1,833 were duplicates); 7,627 records were excluded as a result of screening at title and abstract stage. Reasons for exclusion of 293 records at full-text screening are shown in Figure 1. Five records were unobtainable at full text and therefore excluded [31]–[35]. Reference and citation tracking identified a further 12 eligible records, providing 219 records for narrative analysis (five in Russian, three Japanese and the remainder English). After exclusion of multiple reporting (n = 10) 209 individual studies met review eligibility criteria [11], [12], [36]–[252]; 16 pertained to vaccines against the 2009 influenza A(H1N1) pandemic virus [39], [43], [48], [55], [79], [86], [104], [170], [181], [188], [212], [223]–[225], [232], [243].

Characteristics of the eligible studies are summarised in Table 3. These data have not been presented for each individ ual study due to the volume of data extracted but are available on request. Of note is the large quantity of available data from non-randomised controlled trials (n = 137) and non-randomised clinical studies (n = 43) in addition to the limited data available from countries in medium or low categories of the UN HDI (n = 3). Sub-analysis by resource setting was therefore abandoned due to insufficient data. Immunocompromise due to human immunodeficiency syndrome (HIV), cancer and transplant were approximately equally represented with over 50 studies each, together accounting for more than three quarters (78%) of aetiological groupings.

**Table 3:** Summary of study characteristics (n = 209).

*Characteristic*	*Number of studies*
*Study design*	
Systematic reviews ± meta-analyses	3
Randomised controlled trials	23
Non-randomised controlled trials	137
Non-randomised clinical studies	43
Prospective cohort studies	1
Case series	2
*Setting of conduct*	
Community or primary care	5
Outpatient department or hospital clinic	127
Other	3
Not stated	74
*UN inequality-adjusted Human Development Index 2010*	
Very high	186
High	16
Medium	3
Low	0
No data	4
*Study population (aetiology of immunocompromise)* *	
Human immunodeficiency virus (HIV) infection	58
Cancers	56
Transpla nt recipients	52
Autoimmune diseases receiving immunosuppressive therapy	34
Respiratory diseases receiving immunosuppressive therapy	5
Other	7

*Three studies recruited multiple groups of immunocompromised patients [52], [89], [119].

A median of sixty immunocompromised patients received active influenza vaccination across the 209 studies (interquartile range [IQR] 36 to 110). Studies typically administered the vaccine by intramuscular injection (n = 138) with a minority utilising intradermal (n = 20), subcutaneous (n = 2) and intranasal routes (n = 3). Forty-nine studies did not report these data (most likely intramuscular) and three studies used multiple routes of administration. The median intervention group size for included RCTs was 55 ( IQR 26 to 103) and the median placebo or no vaccination group size was 24 (IQR 17 to 56). A median of 65 vaccinated immunocompromised subjects were recruited (IQR 40 to 116) and sources of funding were declared by 114 studies.

### Risk of bias within studies

Figure S1 summarises the assessment of risk of bias for 191 included experimental studies and prospective cohort designs. The majority of studies were judged at high risk of bias for sequence generation and allocation concealment domains. However, this finding is largely explained by only 23 RCTs meeting the protocol eligibility criteria. Only 10% of RCTs were at high risk of bias due to sequence generation or allocation concealment issues, although risk of bias was unclear in the majority (60% and 80% respectively). Risk of bias due to blinding of study participants, personnel and outcome assessors reduced from 22% in all studies to 5% in RCTs.

Table S2 summarises the assessment of risk of bias for two included case series [48], [232]. Bate *et al* (2010) scored highly within the reporting domain of the risk of bias tool whilst Vazquez-Alvarez *et al* (2010) scored poorly due to limited description of the study characteristics including potential confounding variables. Both studies scored poorly for external and internal validity.

Table S3 summarises the assessment of risk of bias for three included systematic reviews [11], [12], [42]. Risk of bias in all three studies was generally low, although Atashili *et al* (2006) did not assess quality and validity for included studies. Anema *et at* (2008 ) and Atashili *et al* (2006) conducted meta-analyses for a pooled estimate of the effectiveness of influenza vaccination in preventing ILI or laboratory confirmed infection in HIV patients, both including three prospective studies [190], [222], [247] while Atashili *et al* (2006) additionally included a case control study [253].

### Synthesis of results

Outcomes for all 209 individual studies cannot be presented due to volume of data. We pooled studies for analyses according to review questions, irrespective of aetiology of immunocompromise (see discussion for comments on clinical heterogeneity). We identified 47 studies reporting data pertaining to the prevention of influenza-like illness, 16 on prevention of laboratory confirmed influenza, 189 on immune response to vaccination and 152 on adverse events or safety. Of these, we identified 6, 2, 12, and 11 studies respectively which reported outcomes pertaining to vaccines against the 2009 influenza A(H1N1) pandemic virus.

#### Influenza-like illness

**Figure 2:**
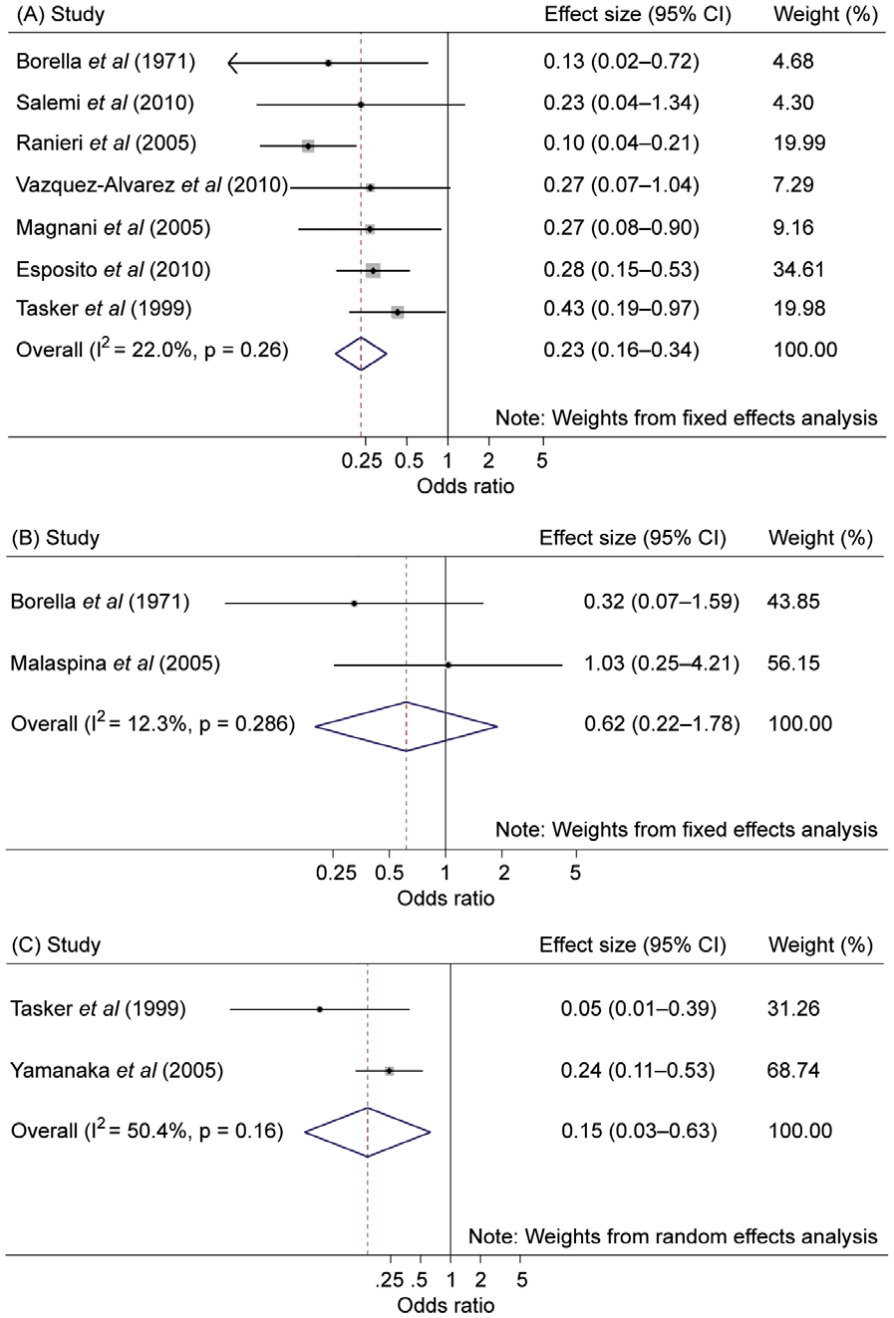
Forest plot for studies on influenza-like illness and laboratory confirmed influenza. Legend: (A) = influenza-like illness (placebo or no vaccination comparator); (B) = influenza-like illness (vaccinated immunocompetent controls); (C) = laboratory confirmed influenza (placebo or no vaccination comparator). Note that each of the three plots shown has different scaled x-axes.

Meta-analysis pooled seven studies of ILI reported in vaccinated immunocompromised patients compared to PNV [59], [97], [159], [190], [202], [222], [232]. Figure 2 shows a pooled effect size of 0.23 (95% CI 0.16 to 0.34; p<0.001) with low statistical heterogeneity (I^2^ = 22.0%; p = not significant [NS]). Meta-analysis also pooled two studies of ILI reported in vaccinat ed immunocompromised patients compared to VICT [59], [160]. Figure 2 shows a pooled effect size of 0.62 (95% CI 0.22 to 1.78; p = NS) with low statistical heterogeneity (I^2^ = 12.3%; p = 0.286).

Two earlier meta-analyses considered vaccination impact on incidence of ILI in immunocompromised patients. Atashili *et al* (2006) pooled one RCT, two non-randomised studies and one case-control study of ILI or laboratory confirmed influenza compared to PNV, estimating a risk difference of −0.27 (95% CI −0.11 to −0.42; p = 0.004) but with significant heterogeneity (I^2^ = 76.8%; p = 0.003) [12]. To address methodological concerns Anema *et al* (2008) performed the same analysis excluding the case-control study, finding a risk ratio of 0.34 (95% CI 0.18 to 0.64; p = <0.001) again with significant heterogeneity (I^2^ = 73%; p = 0.02) [42].

Of those studies unsuitable for meta-analysis, we identified 22 interventional studies and one observational design where no cases of ILI were found in vaccinated immunocompromised patients (including two RCTs with a PNV comparator and 13 non-randomised studies with VICT controls). The remaining studies we identified typically showed a low incident case number of cases, with some noteworthy exceptions. Cumulative incidence of ILI in vaccinated renal transplant recipients immunosuppressed with azathioprine is reported as 5.4% and 8.3% for those on mycophenolate mofetil, compared to 8.1% in healthy controls [204]. The number of upper respiratory tract infections in vaccinated paediatr ic cancer patients completing therapy within six months of randomisation was 0.52±0.79 (mean ± standard deviation), compared to 2.73±1.49 in unvaccinated patients [97]. The inter-group difference reduced to 0.46±0.73 in patients off therapy for 6–24 months, compared to 0.69±0.73 in unvaccinated patients.

#### Laboratory confirmed influenza

Meta-analysis pooled two studies in vaccinated subjects with HIV compared to PNV [222], [247]. Figure 2 shows a pooled effect size of 0.15 (95% CI 0.03 to 0.63; p = 0.01) with moderate statistical heterogeneity (I^2^ = 50.4%; p = NS).

We found limited data from non-randomised studies showing very low numbers of incident cases of laboratory confirmed influenza post vaccination in immunocompromised patients. Nine studies reported no cases during follow-up, and two studies found a single case each. A study by Tasker and colleagues [222] reported a protective efficacy of symptomatic laboratory confirmed influenza A of 100% (95% CI 73% to 100%) in HIV patients compared to PNV controls.

#### Immune response to vaccination

Data on immune response to vaccination for each influenza subtype were pooled for meta-analyses based on CHMP definitions of seroconversion or seroprotection [24] and are summarised in Table 4 with the associated forest plots provided in Figure S2. Table 4 lists several highly significant pooled effects, although moderate to important levels of statistical heterogeneity were typically present. Seroconversion (SC1) with a PNV comparator group was more likely in patients receiving immunologically active vaccine, although statistically significant only for influenza B. Odds of seroconversion (SC2) following vaccination against seasonal A(H1N1) and A(H3N2) were statistically equivalent between immunocompromised patients and VICT cont rols, although the likely estimate of effect suggests an inferior response in patients. Vaccination against pandemic A/H1N1/California/7/2009 resulted in lower but non-significant odds of seroprotection compared to VICT controls although the two pooled studies gave an adjuvanted [60] and non-adjuvanted [154] vaccine in different populations, with significant statistical heterogeneity.

**Table 4:** Summary of meta-analyses for immune response to vaccination.

*Outcome measure*	*Influenza subtype*	*Comparator*	*Number of studies*	*Pooled ES (95% CI)*	*p value of ES*	*I^2^ (%)*	*p value of I^2^*
SC1	A(H1N1) (S)	VICT	50*	0.55 (0.43 to 0.71)	<0.001	53.2	<0.001
SC1	A(H3N2)	VICT	47*	0.55 (0.41 to 0.73)	<0.001	66.9	<0.001
SC1	B	VICT	44*	0.48 (0.36 to 0.62)	<0.001	54.3	<0.001
SC1	A(H1N1) (S)	PNV	3	3.90 (0.42 to 36.64)	NS	77.8	0.01
SC1	A(H3N2)	PNV	3	10.93 (0.92 to 129.80)	NS	82.5	0.003
SC1	B	PNV	2	9.17 (1.05 to 79.97)	0.05	72.7	NS
SC2	A(H1N1) (S)	VICT	6	0.65 (0.39 to 1.09)	NS	13.6	NS
SC2	A(H3N2)	VICT	8	0.60 (0.25 to 1.43)	NS	63.9	0.007
SC2	B	VICT	8	0.42 (0.19 to 0.94)	0.04	69.8	0.002
SP	A(H1N1) (P)	VICT	2	0.22 (0.02 to 2.75)	NS	80.4	0.02
SP	A(H1N1) (S)	VICT	37*	0.36 (0.26 to 0.51)	<0.001	56.9	<0.001
SP	A(H3N2)	VICT	35*	0.39 (0.26 to 0.59)	<0.001	64.1	<0.001
SP	B	VICT	37*	0.37 (0.25 to 0.53)	<0.001	65.1	<0.001

* = some studies contributed two sets of data included in this meta-analysis; (S) = seasonal; (P) = pandemic; ES = effect size; CI = confidence interval; SC1 = seroconversion (≥4 fold rise post vaccination); SC2 = seroconversion (<1∶40 to ≥1∶40 haemagglutination inhibition titre); SP = seroprotection (≥1∶40 haemagglutination inhibition titre post vaccination); VICT = vaccinated immunocompetent controls; PNV = placebo or no vaccination; NS = not statistically significant. See *Figure S2* for citation details.

Of the 85 studies unsuitable for meta-analysis reporting rates of seroconversion, seroprotection or mean geometric increase in HI titre based on serology within 2–4 weeks, many were single-arm but broadly supported the above findings. Notably, statistically equivalent rates of seroconversion (SC1) were found to influenza A(H3N2) and B in patients with primary immunodeficiency [229] and to pandemic A/H1N1/California/7/2009 in paediatric cancer patients [43], both compared to VICT controls. Similar findings were observed in studies comparing seroprotection rates [120], [158]. Further to those subject to meta-analysis only one study reporting serological data with a PNV comparator was identified, but this RCT lacked sufficient description of the control group to permit interpretation [58]. Most studies reporting GMT showed vaccinating immunocompromised patients was associated with a ≥2.5 fold rise, as per CHMP assessment criteria [254]. Immune response among cancer patients vaccinated against pandemic A/H1N1/California/07/2009 using adjuvanted [43] and unspecified [243] vaccines was statistically comparable to that of immunocompetent controls.

#### Adverse events and safety

Adverse event data were unsuitable for meta-analysis owing to difficulty in accurately identifying cases and denominator counts, and potential for bias due to *post hoc* selection of reported outcomes. Local and systemic adverse events were mapped to CHMP criteria [24] in 34 studies. These were generally self-reported, using diary cards or telephone follow-up. Where feasible median adverse event rates were calculated (see Table 5), in addition six studies each reported <3 cases of fever.

**Table 5:** Median adverse event rate by CHMP criteria.

*Adverse event*	*IC patients (%)*	*VICT controls (%)*	*PNV controls (%)*
*Local*			
Ecchymosis	3.1 (2.0 to 4.2; n = 2)	0.0 (0.0 to 0.0; n = 1)	–
Induration	18.9 (10.2 to 30.0; n = 5)	11.0 (6.3 to 15.0; n = 3)	–
*System ic*			
Fever	7.1 (0.0 to 23.3; n = 14)	5.0 (0.0 to 16.7; n = 5)	10.2 (10.0 to 10.3; n = 2)
Malaise	23.6 (0.8 to 44.0; n = 8)	12.0 (0.0 to 25.9; n = 5)	22.1 (20.0 to 24.1; n = 2)
Shivering	10.2 (10.2 to 10.2; n = 1)	16.3 (16.3 to 16.3; n = 1)	–

Values in parentheses show the reported range of adverse events and number of studies; IC = immunocompromised; VICT = vaccinated immunocompetent; PNV = placebo or no vaccination.

Eighty-seven studies reported clinical or laboratory markers of vaccination impact on the underlying immunosuppressive condition. These included CD4+ count and HIV load in HIV-positive patients, relapse and complication rate in cancer patients, allograft rejection rate in transplant patients, disease activity scores in patients with autoimmune conditions and lung function tests in respiratory patients. We did not identify consistent evidence of disease progression or worsening of clinical symptoms related to underlying immunosuppressive condition following vaccination.

Incidence of serious adverse events was reported in 21 studie s, although only 11 of these included a control group. Three hospitalisations occurred in patients with HIV [136] and one transient ischaemic attack in a separate study, which did not specify whether the case was HIV-positive or a healthy control [94]. Madan *et al* (2008) report biopsy-proven allograft rejection within six months of vaccination in four paediatric liver transplant recipients [158]. None of these events (nor those described in other eligible studies) was deemed due to influenza vaccination [43], [94], [136], [158]. Five of 54 paediatric cancer patients developed fever within 48 hours of receiving an adjuvanted vaccination for influenza A/H1N1/California/07/2009. However, whether this was a consequence of vaccination, underlying cancer or concomitant chemotherapy or infection was indeterminable [48].

### Risk of bias across studies

Risk of publication bias was assessed using Begg's funnel plot and confirmed statistically where feasible using Egger's test. There was no evidence of biased reporting among studies subject to meta-analysis.

## Discussion

### Summary of evidence

This systematic review is the first to consider clinical and serologic outcomes following influenza vaccination in immunocompromised patients, incorporating data from the 2009 pandemic period. Our results suggest that vaccinating immunocompromised patients against influenza provides clinical protection from influenza-like illness and laboratory confirmed infection compared to placebo or no vaccination, and the rate of symptomatic disease is comparable to that observed in vaccinated healthy controls. The pooled odds of seroconversion were consistently higher in vaccinated patients compared to PNV controls, although statistical superiority was demonstrated only for influenza B. Conversely, the odds of seroconversion (SC1) and seroprotection conferred by vaccination were consistently and significantly lower in patients compared to VICT controls for seasonal influenza A(H1N1), A(H3N2) and B (see Table 4). The data reviewed offer no consistent evidence of safety concerns, disease progression or serious adverse events following influenza vaccination in immunocompromised populations. Table 5 suggests a higher median rate of malaise in vaccinated immunocompromised patients compared to VICT controls (23.6% vs 12.0%), however malaise is also elevated in PNV controls implying an association with the underlying immunocompromised state.

### Limitations

#### Risk of bias and confounding

Many of the 209 eligible studies were at unclear or high risk of bias across most domains and the number of RCTs was relatively small (n = 23). The majority of studies (n = 137) were non-randomised trials that included a control group, but without robust randomisation selection bias between study arms cannot be excluded. Non-randomised designs may also introduce unbalanced confounding variables, and given that analyses were commonly reported unadjusted, these may reasonably influence the reported effect sizes for each pooled outcome measure. Potential confounders were anticipated and specified in the study protocol. Included cases series conducted during the 2009 influenza A(H1N1) pandemic are likewise at high risk of selection bias. Stratification of meta-analyses by risk of bias was unfeasible due to concerns with selecting a specific domain for classifying studies as ‘low’ or ‘high’. Adverse event data pr esented in Table 5 do not take account of numerous studies broadly stating absence of adverse events in vaccinated groups, potentially introducing reporting bias.

#### Heterogeneity

Moderate to high levels of statistical heterogeneity were present in many of the reported meta-analyses, reaching significance on numerous occasions. Even where effect sizes are consistent, clinical heterogeneity may continue to challenge the validity of meta-analysis. Potential confounders related to aetiology of immunocompromise or intervention characteristics may be responsible for such heterogeneity. This includes pooling of data arising from the 2009 influenza A(H1N1) pandemic vaccine (commonly monovalent, sometimes adjuvanted) with seasonal vaccines although <10% of studies overall involved such vaccines, only one study was included in the ILI meta-analysis (PNV comparator), and the two studies reporting data on prevention of laboratory confirmed influenza offered narrative information only. Previous exposure to influenza vaccination, timing of vaccine administration (in relation to changes in administration of immunosuppressive therapy or disease state) an d immunosenescence may also be important effect modifiers contributing to heterogeneity between the reported outcome measures. Similarly, matching between vaccine and wild type influenza strains is likely to introduce a degree of inter-seasonal variability; however, this does not affect our conclusions in terms of public health policy as these are typically designed to provide consistent advice over multiple seasons. Our analyses reported separately by aetiology of immunocompromise provide a degree of sensitivity testing for pooled results.

#### Other limitations

Paucity of data limited or prevented some analyses. There were insufficient data to adequately report on seroconversion or seroprotection with a PNV comparator. The planned sub-analysis of evidence from resource-poor countries was abandoned due to insufficient data arising from this setting. In addition, it is now recognised that a large proportion of the population aged ≥55 years probably had some degree of pre-existing immunity to the 2009 influenza A(H1N1) pandemic strain, adding further difficulty to the interpretation of data from the pandemic period [255]. We recognise CHMP criteria for serological response to vaccination are based on healthy volunteers aged 18 to 60 years thus may not reflect expected rates of clinical protection observed in vaccinated immunocompromised populations [256].

### Implic ations for public health practice

Our data favour a policy of routinely recommending influenza vaccination to immunocompromised patient groups, who may be at higher risk of influenza and its complications [14], [257]. Many authorities, such as the UK Joint Committee on Vaccination and Immunisation and the US Advisory Committee on Immunization Practices, already recommend vaccinating immunocompromised patients and household or close contacts against influenza to minimise transmission [14], [257]. However, uptake of this intervention is currently unclear but, where data exist, these suggest sub-optimal coverage [258]. Although our findings indicate some mild and self-limiting adverse effects following vaccination, policies should acknowledge these may occur with greater frequency in certain patient groups, and make suitable provision for clinical discretion. Management of infection in immunocompromised patients can be complicated by limited effectiveness of pharmacological therapies and vaccination carries the additional benefit of mitigating emergence of resistance to antiviral agents [9].

### Implications for further research

Methodological limitations affecting the current evidence base mandates new robust studies assessing the incidence of ILI and laboratory confirmed influenza in vaccinated immunocompromised patients. Similarly, robust studies are needed to inform revised CHMP seroconversion and seroprotection criteria applicable to immunocompromised patients. Further primary research is warranted to quantify factors contributing to heterogeneity, including the utility of second ‘booster’ doses, immunological adjuvants and degree of immunosuppression on rates of clinical protection and response to vaccination. Systematic reviews and meta-analyses are indicated to assess the impact of vaccinating immunocompromised patients on influenza-related morbidity and mortality. In addition, resource poor countries should be supported to conduct robust studies of influenza vaccination in their immunocompromised populations. Proportionally different comorbidities such as malnutrition or co-in fection with HIV may be encountered and response to vaccination among indigenous groups and ethnic minorities may differ in these settings compared to developed countries.

### Conclusion

Our systematic review and meta-analyses suggest immunocompromised patients do manifest an immune response to vaccination that, while not as vigorous as that of healthy controls, probably confers a similar level of clinical protection against influenza and, importantly, does so without causing excess harm. Limitations including potential for bias and confounding and the presence of statistical or clinical heterogeneity mean the evidence for these assertions is generally weak, but the direction of effects are remarkably consistent. Nevertheless, our study supports national and international public health policy recommendations for the targeting of immunocompromised patients for influenza vaccination.

## Supporting Information

Figure S1 **Summary of risk of bias using the Cochrane Collaboration tool (n = 191). Legend: green = low risk of bias; yellow = unclear risk of bias; red = high risk of bias.**
(PDF)

Figure S2 **Forest plots for immune response to vaccination question.** *Figure S2.1*. Forest plot of studies of seroconversion (≥4 fold rise in haemagglutination inhibition titre): seasonal influenza A(H1N1), vaccinated immunocompromised patients versus vaccinated immunocompetent controls. *Figure S2.2*. Forest plot of studies of seroconversion (≥4 fold rise in haemagglutination inhibition titre): influenza A(H3N2), vaccinated immunocompromised patients versus vaccinated immunocompetent controls. *Figure S2.3*. Forest plot of studies of seroconversion (≥4 fold rise in haemagglutination inhibition titre): seasonal influenza B, vaccinated immunocompromised patients versus vaccinated immunocompetent contr ols. *Figure S2.4*. Forest plot of studies of seroconversion (≥4 fold rise in haemagglutination inhibition titre): seasonal influenza A(H1N1), vaccinated immunocompromised patients versus placebo or no vaccination. *Figure S2.5*. Forest plot of studies of seroconversion (≥4 fold rise in haemagglutination inhibition titre): influenza A(H3N2), vaccinated immunocompromised patients versus placebo or no vaccination. *Figure S2.6*. Forest plot of studies of seroconversion (≥4 fold rise in haemagglutination inhibition titre): influenza B, vaccinated immunocompromised patients versus placebo or no vaccination. *Figure S2.7*. Forest plo t of studies of seroconversion (<1∶40 pre-vaccination to ≥1∶40 haemagglutination inhibition titre post vaccination): seasonal influenza A(H1N1), vaccinated immunocompromised patients versus vaccinated immunocompetent controls. *Figure S2.8*. Forest plot of studies of seroconversion (<1∶40 pre-vaccination to ≥1∶40 haemagglutination inhibition titre post vaccination): influenza A(H3N2), vaccinated immunocompromised patients versus vaccinated immunocompetent controls. *Figure S2.9*. Forest plot of studies of seroconversion (<1∶40 pre-vaccination to ≥1∶40 haemagglutination inhibition titre post vaccination): seasonal influenza B, vaccinated immunocompromised patients versus vaccinated immunocompetent controls. *Figure S2.10*. Forest plot of studies of seroprotection (≥1∶40 haemagglutination inhibition titre post vaccination): seasonal influenza A(H1N1), vaccinated immunocompromised patients versus vaccinated immunocompetent controls. *Figure S2.11*. Forest plot of studies of seroprotection (≥1∶40 haemagglutination inhibition titre post vaccination): influenza A(H3N2), vaccinated immunocompromised patients versus vaccinated immunocompetent controls. *Figure S2.12*. Forest plot of studies of seroprotection (≥1∶40 haemagglutination inhibition titre post vaccination): seasonal influenza B, vaccinated immunocompromised patients versus vaccinated immunocompetent controls. *Figure S2< /xref>.13*. Forest plot of studies of seroprotection (≥1∶40 haemagglutination inhibition titre post vaccination): pandemic influenza A(H1N1), vaccinated immunocompromised patients versus vaccinated immunocompetent controls.
(PDF)

Table S1 **MEDLINE search construct.** Legend: PICO = research question in terms of population, intervention, comparators and outcomes. MeSH = Medical Subject Headings (US National Library of Medicine).
(PDF)

Table S2 **Summary of risk of bias using the Downs and Black (1998) tool (n = 2). Legend: N/A = not applicable; higher score = less risk of bias.**
(PDF)

Table S3 **Summary of risk of bias using the US AHRQ tool (n = 3). Legend: Response indicates whether associated elements for reduction of bias have been met.**
(PDF)

Protocol S1 (PDF)

Checklist S1 (DOC)
