# Supplementary figures and images for: Influenza Vaccination for Immunocompromised Patients: Systematic Review and Meta-Analysis from a Public Health Policy Perspective
Source: PLoS One. 2011 Dec 22;6(12):e29249. doi: 10.1371/journal.pone.0029249 (PMC3245259; doi:10.1371/journal.pone.0029249)

**Figure S1. Summary of risk of bias using the Cochrane Collaboration tool (n = 191)**

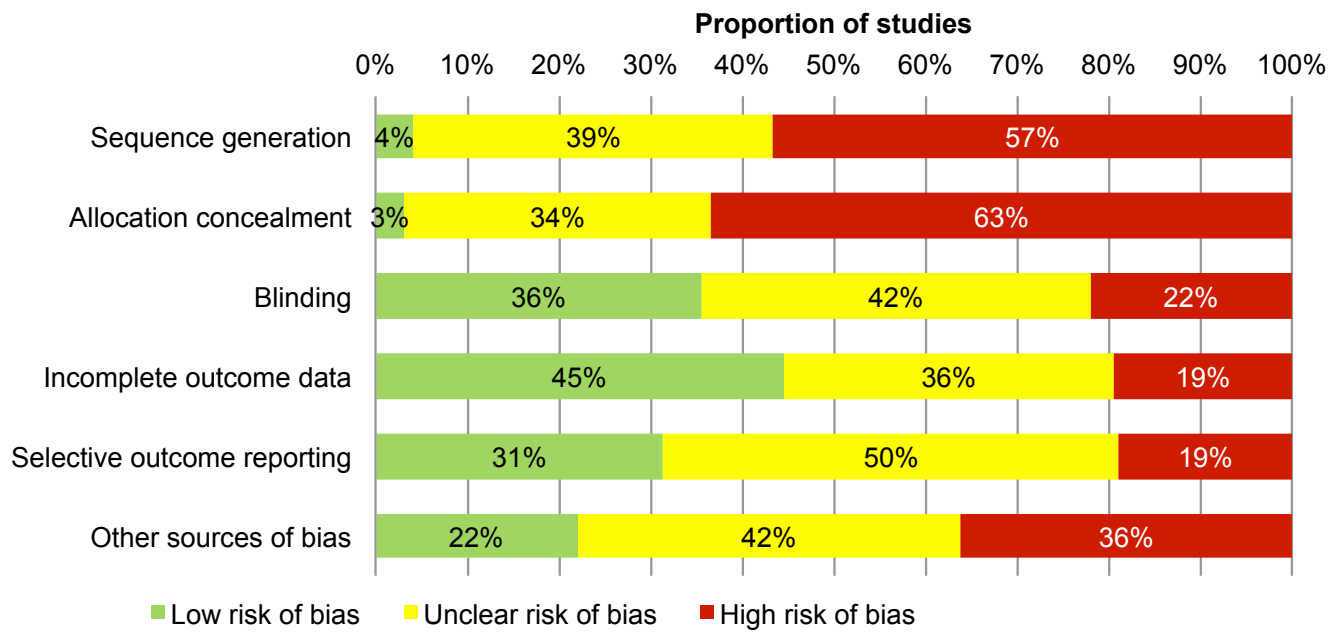

Supplement: Figure S1 — Summary of risk of bias using the Cochrane Collaboration tool (n = 191). Legend: green = low risk of bias; yellow = unclear risk of bias; red = high risk of bias. (PDF) [file pone.0029249.s001.pdf]
