# Supplementary material for: Influenza Vaccination for Immunocompromised Patients: Systematic Review and Meta-Analysis from a Public Health Policy Perspective
Source: PLoS One. 2011 Dec 22;6(12):e29249. doi: 10.1371/journal.pone.0029249 (PMC3245259; doi:10.1371/journal.pone.0029249)

Figure S2. Forest plots for immune response to vaccination question

Figure S2.1. Forest plot of studies of seroconversion (≥4 fold rise in HI titre): seasonal influenza A/H1N1, vaccinated immunocompromised patients versus vaccinated immunocompetent controls

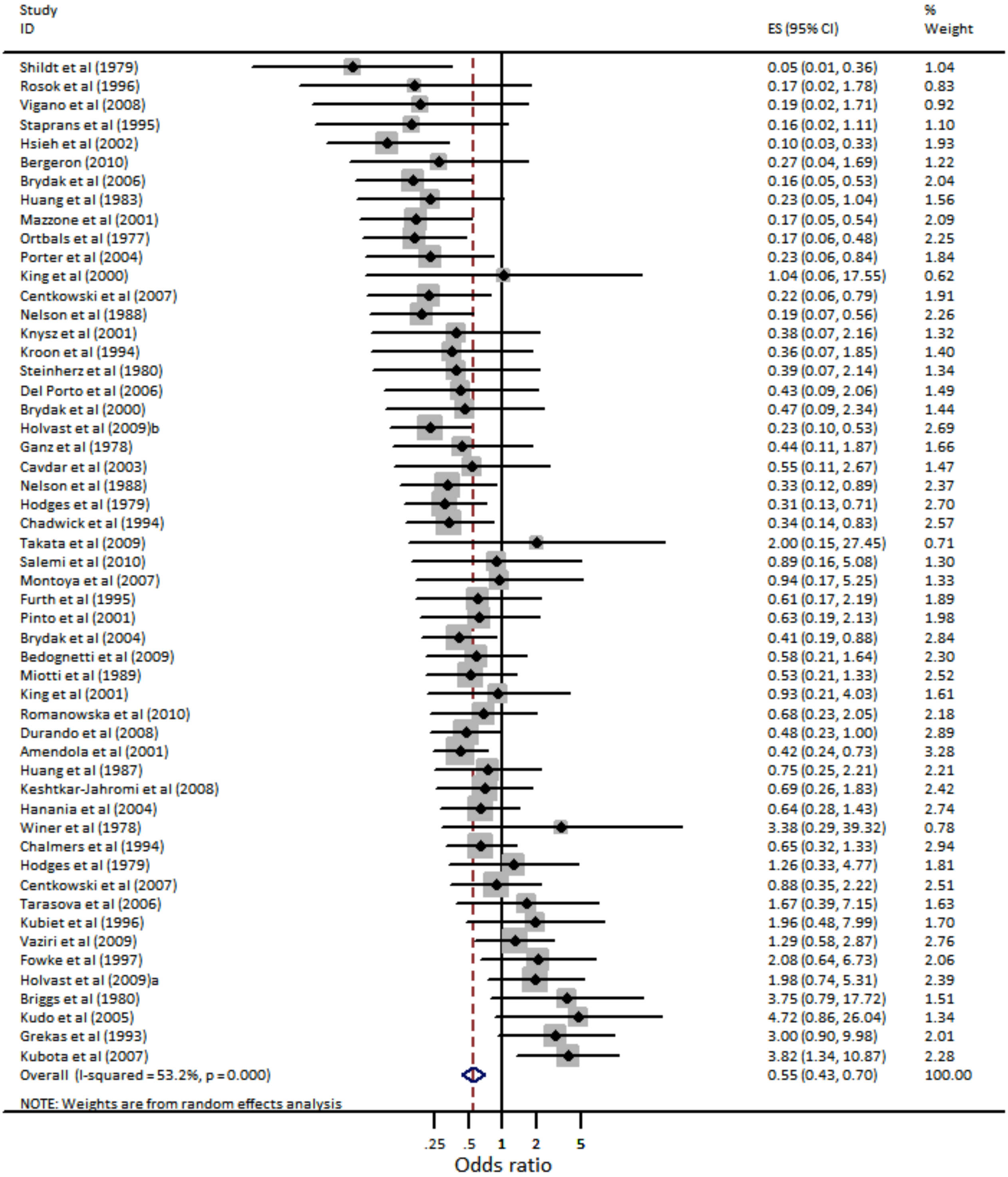

Figure S2.2. Forest plot of studies of seroconversion (≥4 fold rise in HI titre): influenza A/H3N2, vaccinated immunocompromised patients versus vaccinated immunocompetent controls

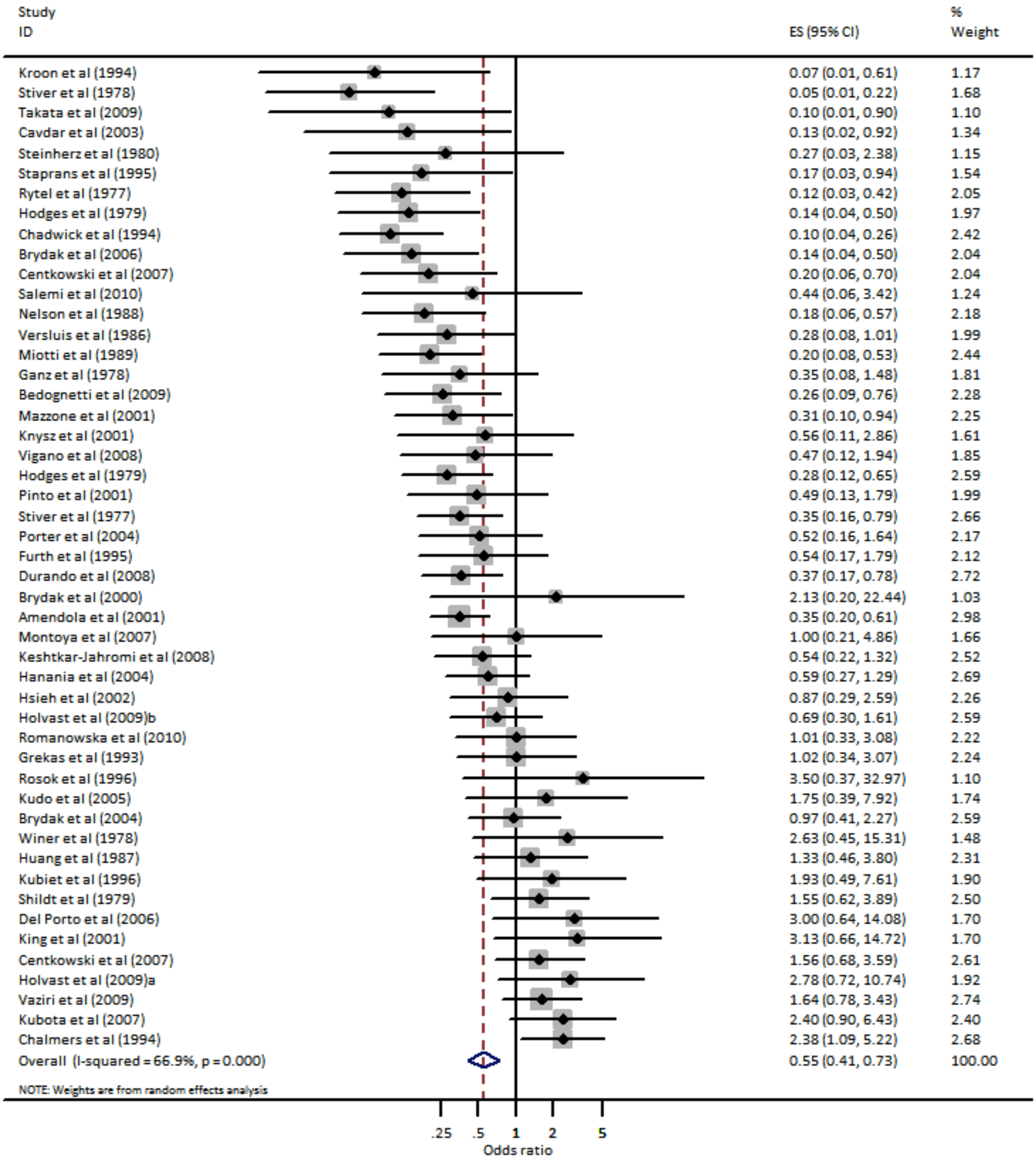

Figure AS2.3. Forest plot of studies of seroconversion (≥4 fold rise in HI titre): seasonal influenza B, vaccinated immunocompromised patients versus vaccinated immunocompetent controls

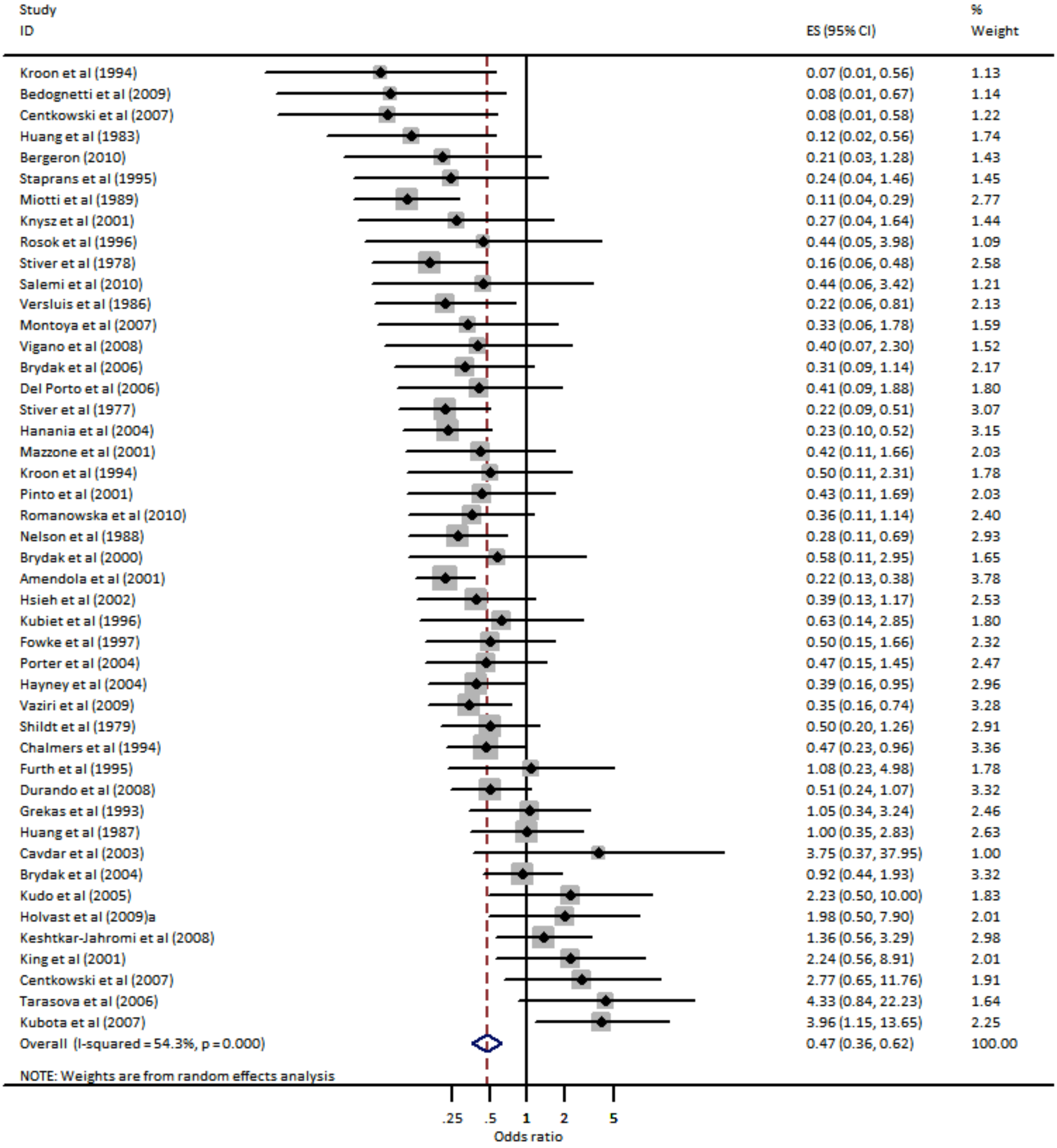

Figure AS2.4. Forest plot of studies of seroconversion (≥4 fold rise in HI titre): seasonal influenza A/H1N1, vaccinated immunocompromised patients versus placebo or no vaccination

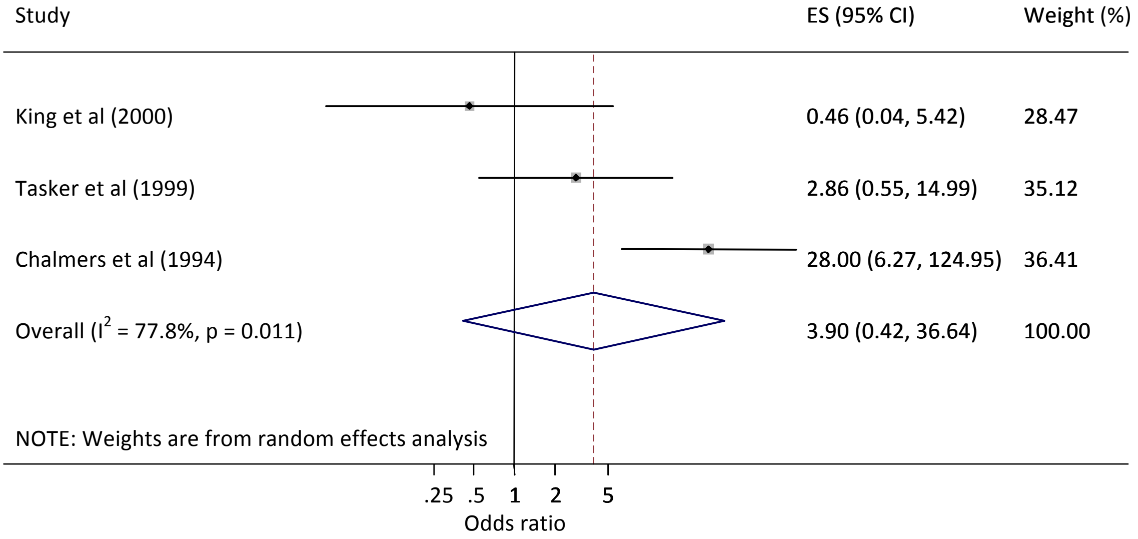

Figure S2.5. Forest plot of studies of seroconversion (≥4 fold rise in HI titre): influenza A/H3N2, vaccinated immunocompromised patients versus placebo or no vaccination

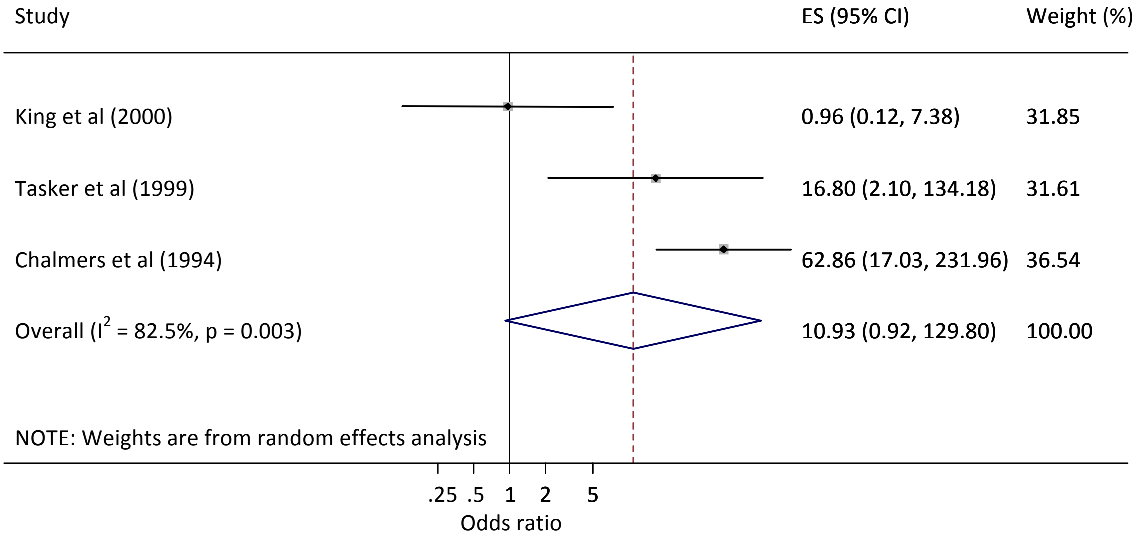

Figure S2.6. Forest plot of studies of seroconversion (≥4 fold rise in HI titre): influenza B, vaccinated immunocompromised patients versus placebo or no vaccination

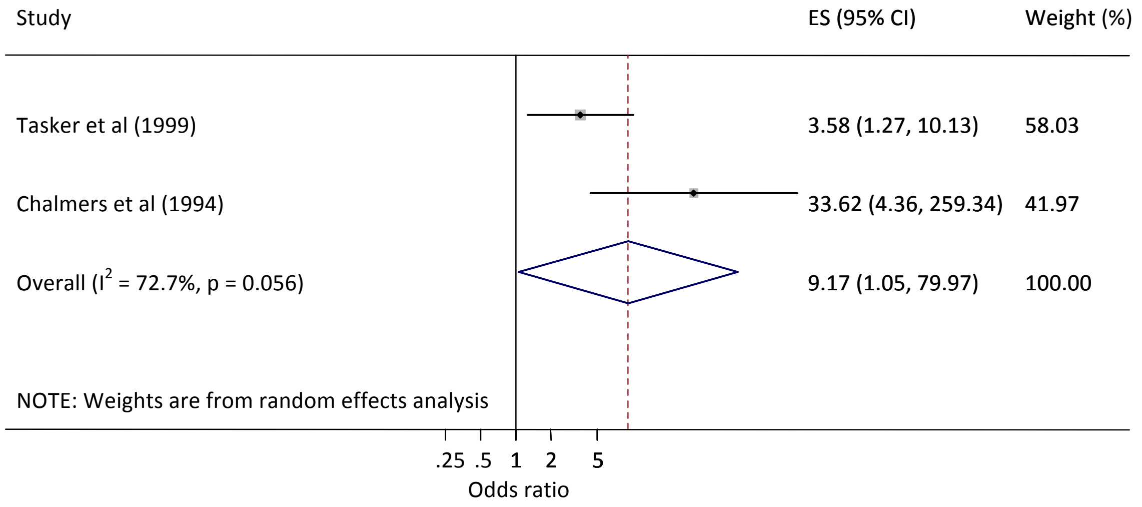

Figure S2.7. Forest plot of studies of seroconversion (<1:40 pre-vaccination to ≥1:40 HI titre post vaccination): seasonal influenza A/H1N1, vaccinated immunocompromised patients versus vaccinated immunocompetent controls

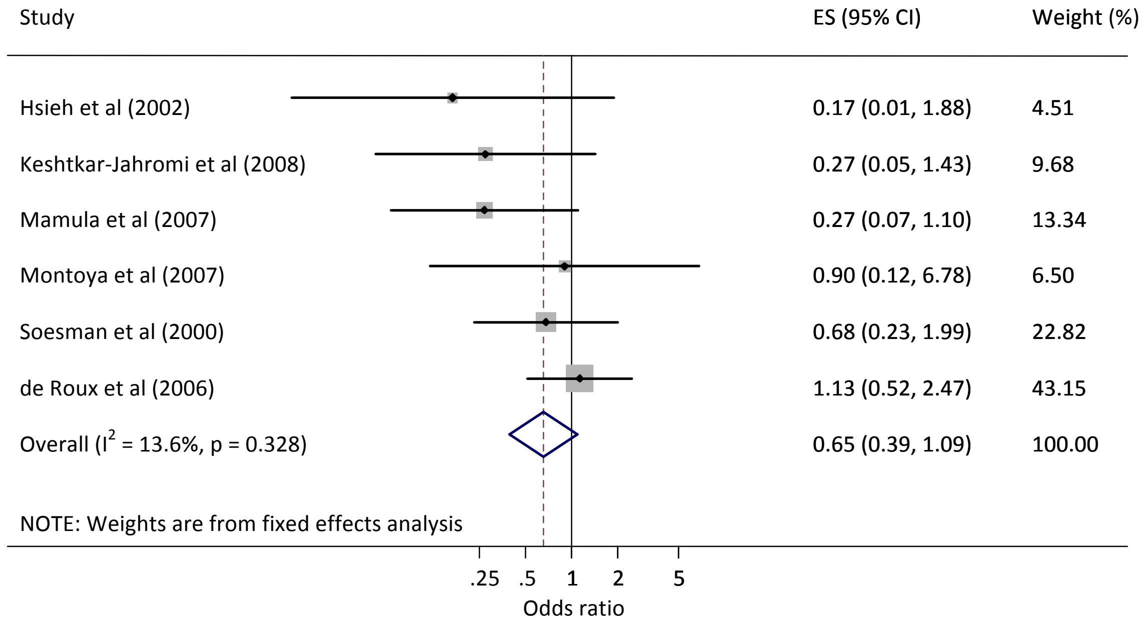

Figure S2.8. Forest plot of studies of seroconversion (<1:40 pre-vaccination to ≥1:40 HI titre post vaccination): influenza A/H3N2, vaccinated immunocompromised patients versus vaccinated immunocompetent controls

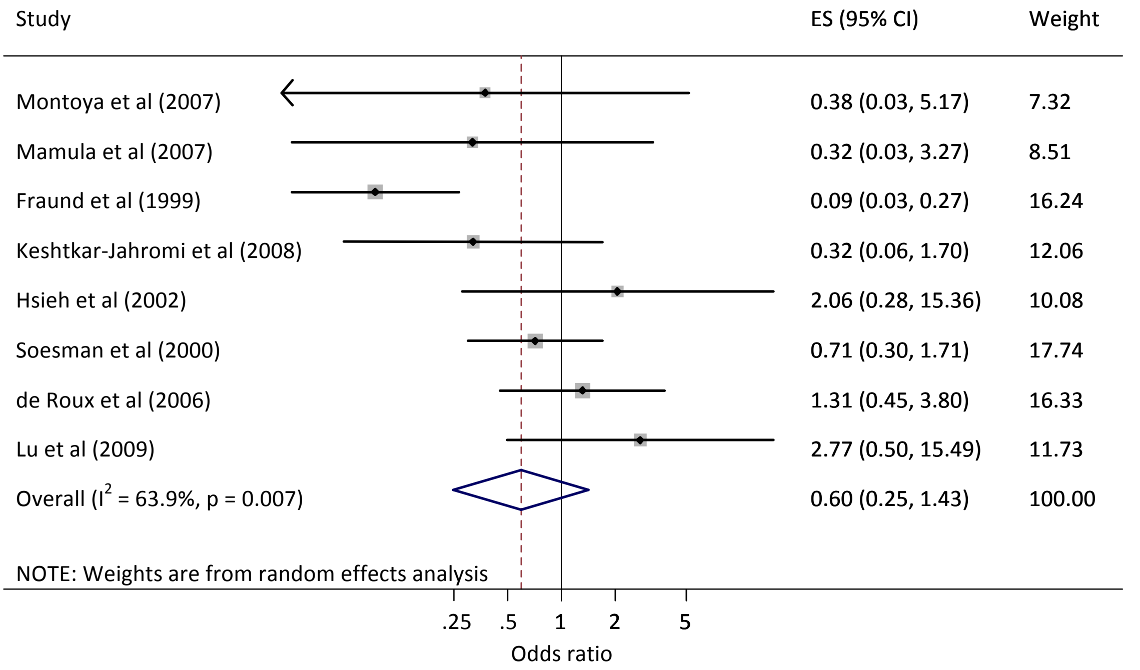

Figure S2.9. Forest plot of studies of seroconversion (<1:40 pre-vaccination to ≥1:40 HI titre post vaccination): seasonal influenza B, vaccinated immunocompromised patients versus vaccinated immunocompetent controls

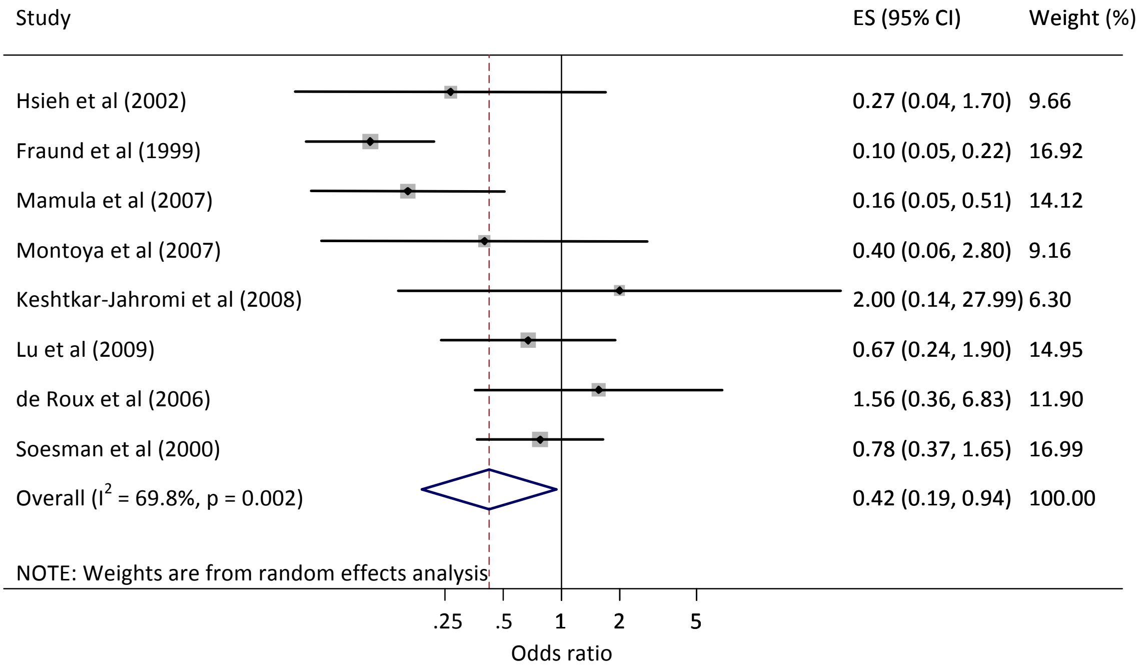

Figure S2.10. Forest plot of studies of seroprotection (≥1:40 HI titre post vaccination): seasonal influenza A/H1N1, vaccinated immunocompromised patients versus vaccinated immunocompetent controls

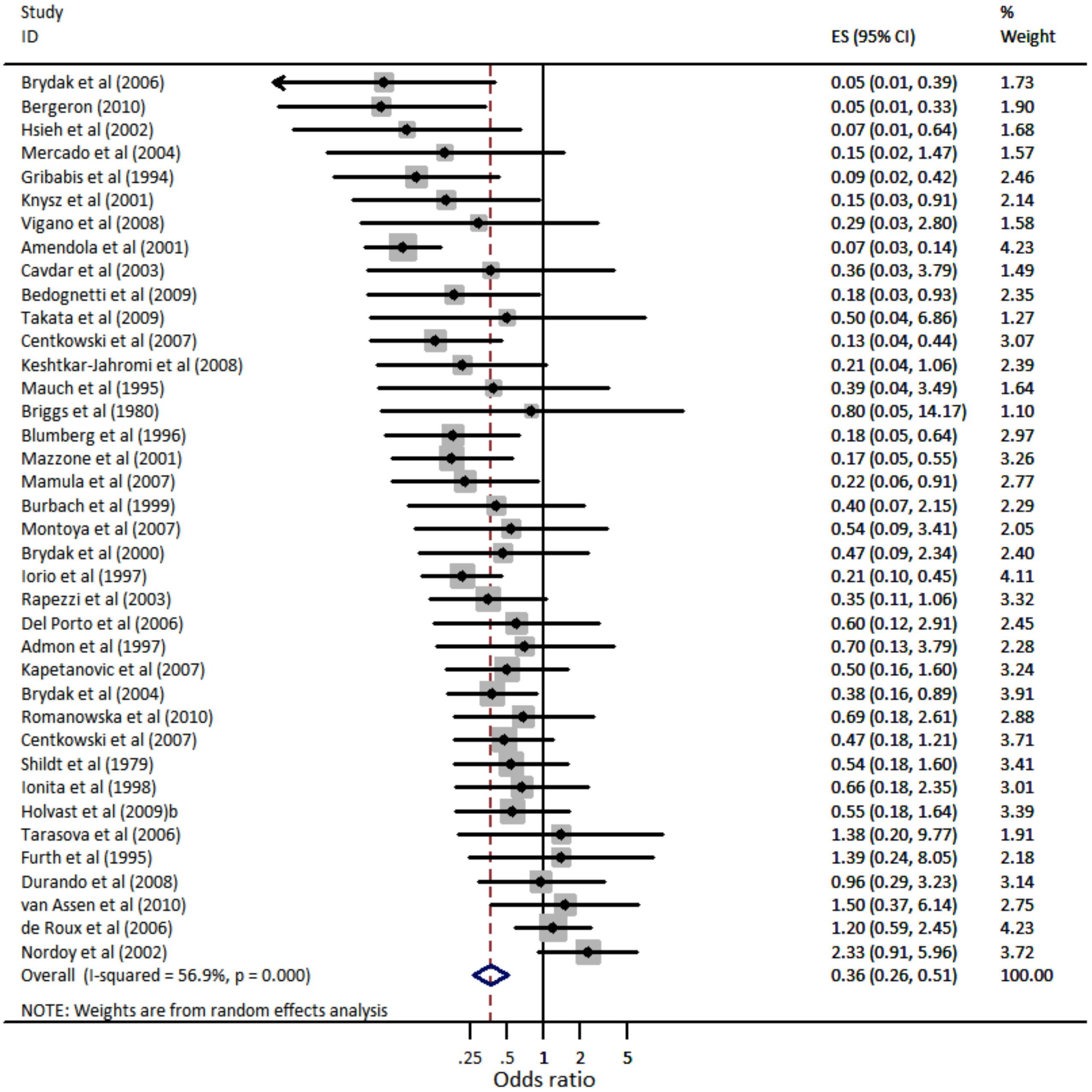

Figure S2.11. Forest plot of studies of seroprotection (≥1:40 HI titre post vaccination): influenza A/H3N2, vaccinated immunocompromised patients versus vaccinated immunocompetent controls

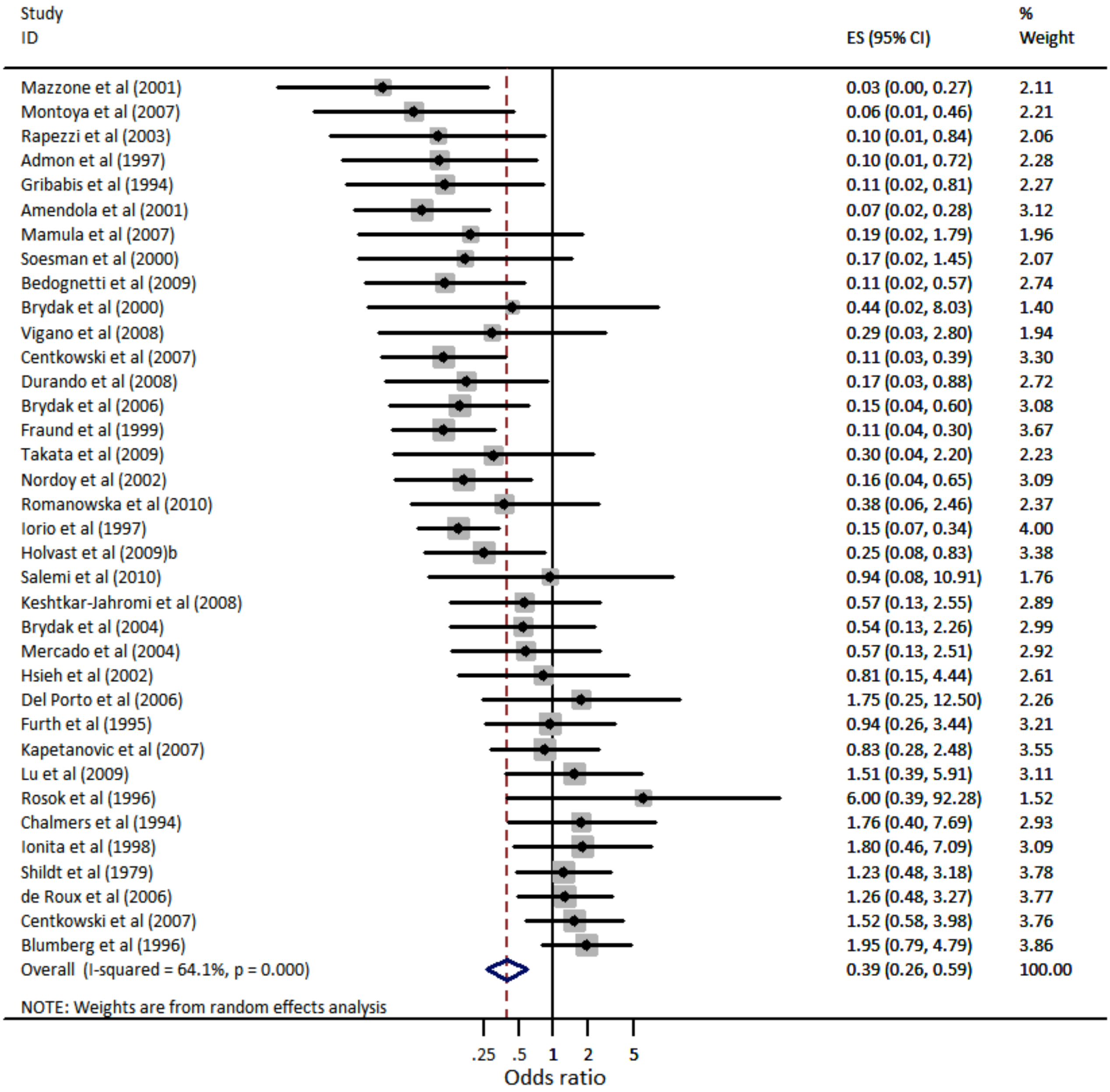

Figure S2.12. Forest plot of studies of seroprotection (≥1:40 HI titre post vaccination): seasonal influenza B, vaccinated immunocompromised patients versus vaccinated immunocompetent controls

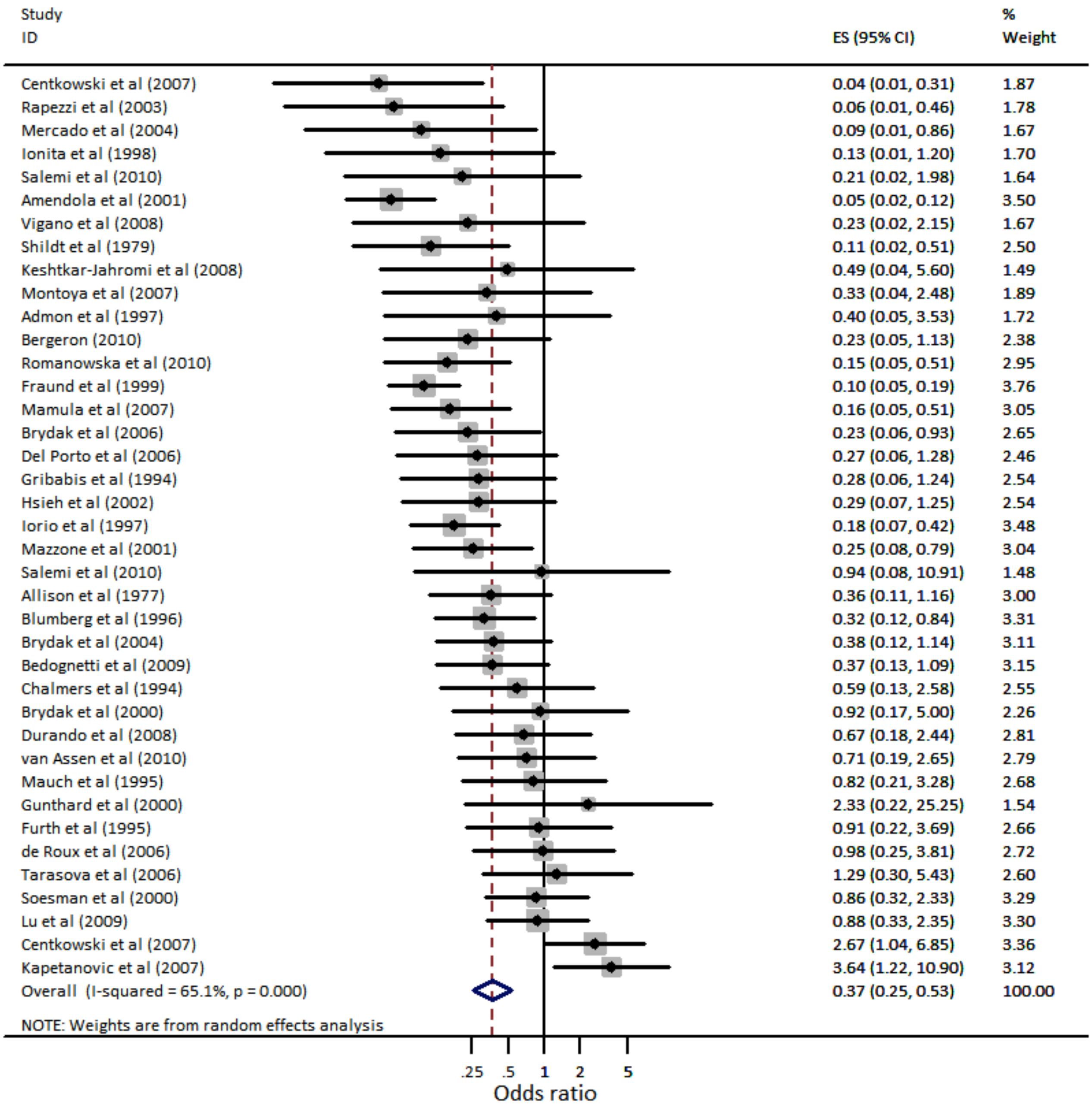

Figure S2.13. Forest plot of studies of seroprotection (≥1:40 HI titre post vaccination): pandemic influenza A/H1N1, vaccinated immunocompromised patients versus vaccinated immunocompetent controls

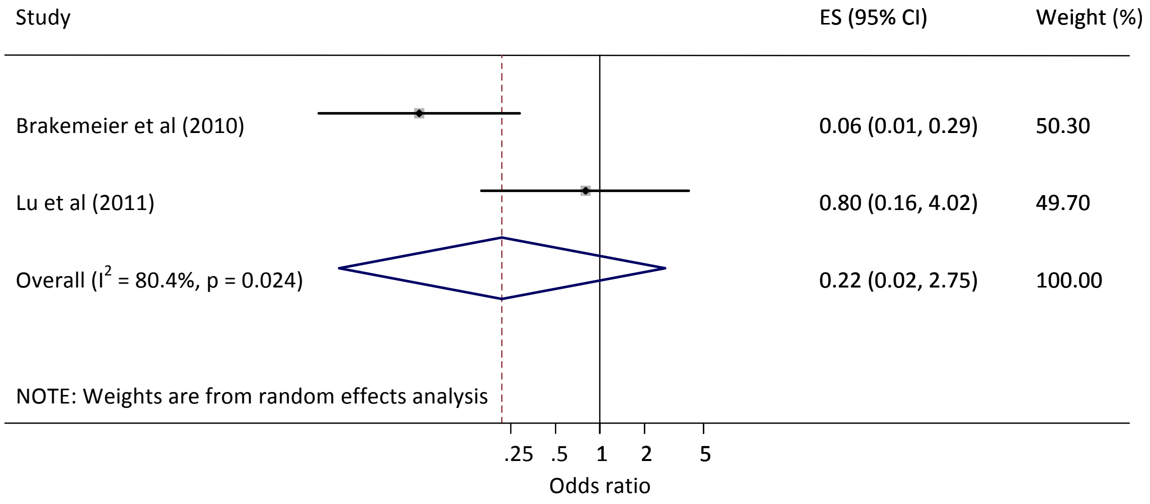

Supplement: Figure S2 — Forest plots for immune response to vaccination question. Figure S2.1. Forest plot of studies of seroconversion (≥4 fold rise in haemagglutination inhibition titre): seasonal influenza A(H1N1), vaccinated immunocompromised patients versus vaccinated immunocompetent controls. Figure S2.2. Forest plot of studies of seroconversion (≥4 fold rise in haemagglutination inhibition titre): influenza A(H3N2), vaccinated immunocompromised patients versus vaccinated immunocompetent controls. Figure S2.3. Forest plot of studies of seroconversion (≥4 fold rise in haemagglutination inhibition titre): seasonal influenza B, vaccinated immunocompromised patients versus vaccinated immunocompetent contr ols. Figure S2.4. Forest plot of studies of seroconversion (≥4 fold rise in haemagglutination inhibition titre): seasonal influenza A(H1N1), vaccinated immunocompromised patients versus placebo or no vaccination. Figure S2.5. Forest plot of studies of seroconversion (≥4 fold rise in haemagglutination inhibition titre): influenza A(H3N2), vaccinated immunocompromised patients versus placebo or no vaccination. Figure S2.6. Forest plot of studies of seroconversion (≥4 fold rise in haemagglutination inhibition titre): influenza B, vaccinated immunocompromised patients versus placebo or no vaccination. Figure S2.7. Forest plo t of studies of seroconversion (<1∶40 pre-vaccination to ≥1∶40 haemagglutination inhibition titre post vaccination): seasonal influenza A(H1N1), vaccinated immunocompromised patients versus vaccinated immunocompetent controls. Figure S2.8. Forest plot of studies of seroconversion (<1∶40 pre-vaccination to ≥1∶40 haemagglutination inhibition titre post vaccination): influenza A(H3N2), vaccinated immunocompromised patients versus vaccinated immunocompetent controls. Figure S2.9. Forest plot of studies of seroconversion (<1∶40 pre-vaccination to ≥1∶40 haemagglutination inhibition titre post vaccination): seasonal influenza B, vaccinated immunocompromised patients versus vac [file pone.0029249.s002.pdf]
