# Supplementary material for: Influenza Vaccination for Immunocompromised Patients: Systematic Review and Meta-Analysis from a Public Health Policy Perspective
Source: PLoS One. 2011 Dec 22;6(12):e29249. doi: 10.1371/journal.pone.0029249 (PMC3245259; doi:10.1371/journal.pone.0029249)
Supplement: Table S1 — MEDLINE search construct. Legend: PICO = research question in terms of population, intervention, comparators and outcomes. MeSH = Medical Subject Headings (US National Library of Medicine). (PDF) [file pone.0029249.s003.pdf]

Table S1. MEDLINE search construct.

PICO	*MeSH thesaurus terms*	*Free text terms*
Population	immunosuppression	immunosuppress* OR immuno-supress*
	immunocompromised host	immunocompromis* OR immuno-compromis*
	immunologic deficiency syndromes	immun* AND deficien*
	acquired immunodeficiency syndrome	immunodeficien* OR immuno-deficien*
		immunoglobulin AND deficien*
		complement AND deficien*
	phagocyte bactericidal dysfunction	phagocyte AND dysfunction*
	HIV	HIV
	tuberculosis	tuberculosis OR TB
	transplants	transplant*
	stem cell transplantation	
	neoplasms	neoplasm* OR cancer
	carcinoma	carcinoma*
	lymphoma	lymphoma*
	leukemia	leukemi* OR leukaemi*
	nutrition disorders	nutrition* AND disorder*
	malnutrition	malnutrition*
	splenic diseases	
	splenectomy	asplenia
	steroids	steroid* OR corticosteroid*
	antineoplastic agents	antineoplastic AND agent*
	chemotherapy, adjuvant	chemotherap*
	cytotoxicity, immunologic	cytotoxic*
	antirheumatic agents	
	immunosuppressive agents	
Intervention	influenza vaccines	(influenza OR flu OR season* OR pandemic OR H1N1) AND (vaccin* OR immunis* OR immuniz* OR inoculat*)
	viral vaccines	(inactiv* OR attenu* OR adjuvant*) AND (vaccin* OR immunis* OR immuniz* OR inoculat*)
	vaccines, inactivated	split AND vir*
	vaccines, attenuated	disrupt* AND vir*

	adjuvants, immunologic	surface AND antigen* AND inactivat*
	immunization	
Comparators	placebos	no AND (vaccin* OR immunis* OR immuniz* OR inoculat*)
		(placebo OR sham) AND (vaccin* OR immunis* OR immuniz* OR inoculat*)
Outcomes	influenza, human	influenza OR flu OR H1N1
	influenza A virus	(influenza-like OR flu-like) OR (influenza AND like) OR (flu AND like) AND illness OR ILI
		diagno* AND (influenza OR flu)
		intention-to-treat OR (intention AND to AND treat) OR ITT
		laboratory AND confirm* AND (influenza OR flu)
		(intention-to-treat AND influenza) OR (intention-to-treat AND flu) OR (intention AND to AND treat AND influenza) OR (intention AND to AND treat AND flu) OR ITTI
	hemagglutination inhibition tests	((haemagglutin* OR hemagglutin*) AND inhibit* AND anti*) AND HAI
	immunoglobulin G	(haemagglutin* OR hemagglutin*) OR HA AND (immunoglobulin G OR IgG) AND antibod*
	immunoglobulin A	(haemagglutin* OR hemagglutin*) OR HA AND (immunoglobulin A OR IgA) AND antibod*
	immunoglobulins	
	antibody formation	
	antibodies	
		(adverse AND event*) OR safe* OR (side AND effect*) or (adverse AND effect*) OR harm*
