# Supplementary material for: Influenza Vaccination for Immunocompromised Patients: Systematic Review and Meta-Analysis from a Public Health Policy Perspective
Source: PLoS One. 2011 Dec 22;6(12):e29249. doi: 10.1371/journal.pone.0029249 (PMC3245259; doi:10.1371/journal.pone.0029249)
Supplement: Table S2 — Summary of risk of bias using the Downs and Black (1998) tool (n = 2). Legend: N/A = not applicable; higher score = less risk of bias. (PDF) [file pone.0029249.s004.pdf]

Table S2. Summary of risk of bias using the Downs and Black (1998) tool (n = 2).

Domain	***Bate* et al *(2010)***	***Vazquez-Alvarez* et al *(2010)***
Reporting (max. score = 11)	11	2
External validity (max. score = 3)	0	0
Internal validity – bias (max. score 7)	5	3
Internal validity – confounding (max. score = 6)	3	2
Power (max. score = 5)	N/A	N/A
