# Supplementary material for: Influenza Vaccination for Immunocompromised Patients: Systematic Review and Meta-Analysis from a Public Health Policy Perspective
Source: PLoS One. 2011 Dec 22;6(12):e29249. doi: 10.1371/journal.pone.0029249 (PMC3245259; doi:10.1371/journal.pone.0029249)
Supplement: Table S3 — Summary of risk of bias using the US AHRQ tool (n = 3). Legend: Response indicates whether associated elements for reduction of bias have been met. (PDF) [file pone.0029249.s005.pdf]

Table S3. Summary of risk of bias using the US AHRQ tool (n = 3).

Domain	***Study***		
	Anema et al (2008)	***Atashili et al (2006)***	***Goossen et al (2009)***
Study question	Yes	Yes	Yes
Search strategy	Yes	Yes	Yes
Inclusion and exclusion criteria	Yes	Partial	Partial
Interventions	Yes	Yes	Yes
Outcomes	Partial	Partial	Yes
Data extraction	Yes	Yes	Partial
Study quality and validity	Partial	No	Yes
Data synthesis and analysis	Yes	Yes	Partial
Results	Yes	Yes	Yes
Discussion	Yes	Yes	Yes
Funding and sponsorship	No	Yes	Yes
