# Supplementary material for: Influenza Vaccination for Immunocompromised Patients: Systematic Review and Meta-Analysis from a Public Health Policy Perspective
Source: PLoS One. 2011 Dec 22;6(12):e29249. doi: 10.1371/journal.pone.0029249 (PMC3245259; doi:10.1371/journal.pone.0029249)
Supplement: Protocol S1 — (PDF) [file pone.0029249.s006.pdf]

Clinical effectiveness of influenza vaccination for immunocompromised patients: a systematic review

Protocol

Health Protection Research Group, Division of Epidemiology and Public Health, School of Community Health Sciences, University of Nottingham, UK

A project commissioned by the World Health Organisation Global Influenza Programme

Prepared by: Dr Charles R. Beck, Dr Bruce C. McKenzie
Document date: 20 February 2011
Version: 3

Senior supervisor: **Professor Jonathan S. Nguyen-Van-Tam**

Project lead: **Dr Charles R. Beck**
Specialty Registrar in Public Health

Room A40h
Clinical Sciences Building
Nottingham City Hospital
Hucknall Road
Nottingham, UK
NG5 1PB

Email: charles.beck@nottingham.ac.uk
Telephone: +44 (0)115 823 1815

Source of funding: **World Health Organization Global Influenza Programme**

Table of Contents

1.0 BACKGROUND **3**
2.0 REVIEW QUESTIONS **4**
3.0 STUDY SELECTION **5**
4.0 ASSESSMENT OF RISK OF BIAS AND DATA EXTRACTION **6**
5.0 DATA SYNTHESIS **8**
6.0 DISSEMINATION **9**
7.0 RESOURCE IMPLICATIONS **9**
8.0 REFERENCES **9**
APPENDIX 1 – LITERATURE SEARCH STRATEGY **11**
APPENDIX 2 – DATA EXTRACTION FORM **18**

1.0 Background

This protocol describes the methodology for undertaking a systematic review to assess the clinical effectiveness of influenza vaccination in immunocompromised patients, which will be conducted and reported according to the requirements of the Preferred Reporting Items for Systematic Reviews and Meta-Analyses (PRISMA) statement.[1] The University of Nottingham has been commissioned by the World Health Organization Global Influenza Programme (WHO GIP) to undertake this work. Background to the systematic review was provided by WHO GIP in the agreement for performance of work (APW) and is reproduced below.

> *Previous to the 2009 influenza pandemic, WHO infection protection and control (IPC) guidelines already existed for standard precautions in healthcare facilities,[2] epidemic- and pandemic- prone acute respiratory diseases,[3] and for avian (H5N1) influenza.[4] During the pandemic, rapid advice IPC guidelines for pandemic (H1N1) 2009 were released.[5] However, these guidelines were developed as rapid advice, interim guidelines or aide-memoirs, without systematic evidence reviews as supporting materials. To assist in the production of a standard infection prevention and control for acute respiratory diseases (ARDs) guideline and a clinical management for severe influenza guideline, systematic reviews of certain areas of literature are required to fill identified evidence gaps. In particular, there is little coverage of the use of vaccination for the protection of higher risk individuals from infection with influenza and other acute respiratory diseases. Some systematic reviews exist, for example for the use of influenza vaccines in HCWs to protect the elderly, and of their use in certain high risk groups such as children with cancer, and patients with cystic fibrosis, COPD and asthma. However, not all higher risk patient groups are encompassed by these reviews, and the focus is mostly on influenza. Therefore, to strengthen the evidence base in preparation for these two upcoming guidelines, two systematic reviews are required. Firstly, a systematic review of the effectiveness of all relevant vaccines against ARDs given to HCWs for the protection of all relevant higher risk patient groups from infection with ARDs. Secondly, a systematic review of vaccination of immunocompromised patients against influenza is required. In this particular patient group, other treatment options may be less effective, therefore prevention of infection through vaccination may be an important aspect of clinical management. In addition, a significant evidence gap exists in terms of the use of IPC measures in different settings, thus*

the reviews should consider the context of the research, with particular emphasis on assessment of efficacy and relative impact in low resource settings.

2.0 Review questions

The objective of this review is to assess the clinical effectiveness of influenza vaccination for the prevention of influenza infection in immunocompromised patients. The subject population of immunocompromised patients includes sub-groups of individuals with reduced immune function due to a number of different aetiologies. The criteria used in this systematic review to identify immunocompromised patients and relevant sub-groups is based on published WHO guidelines and UK Department of Health immunisation policy to prevent influenza infection.[6 7]

WHO identified a significant evidence gap in the use of infection prevention and control measures in different settings. This review will thus consider the context of the identified studies, including an assessment of efficacy and relative impact in low resource settings (although studies conducted in developed countries will be included).

The review questions to inform the objective are:

1. What is the effectiveness of seasonal influenza and 2009 pandemic influenza A(H1N1) vaccination (hereafter referred to as 'vaccination') to prevent clinically diagnosed influenza-like illness in immunocompromised patients?
2. What is the efficacy of vaccination in preventing laboratory confirmed infection in immunocompromised patients?
3. What immunological response is produced after vaccinating immunocompromised patients?
4. What adverse effects are associated with vaccination in immunocompromised patients?

The population, intervention, comparators, and outcomes (PICO) framework to inform the systematic review objectives are presented below.

Population: All persons of any age who are immunocompromised, whether due to primary immunodeficiency (genetic defects) or secondary immunodeficiency (such as HIV infection, malignancy, poor nutritional status or use of immunosuppressive drugs)

Intervention: Seasonal influenza or 2009 pandemic influenza A(H1N1) vaccinations

Comparators: No vaccination or placebo/sham vaccination

Outcomes: Clinically diagnosed influenza or influenza-like illness (ILI; intention-to-treat patients [ITT]), laboratory confirmed influenza (intention-to-treat influenza [ITTI]), immunological response to vaccination, and adverse effects associated with vaccination

3.0 Study selection

The literature search strategy including search terms, limits and sources is shown in Appendix 1. The eligibility criteria for identified studies are produced below.

Inclusion criteria

- Experimental studies or systematic reviews (± meta-analyses) reporting data on the efficacy, effectiveness, immunological response or adverse effects associated with influenza vaccination of immunocompromised patients to prevent infection from seasonal influenza or 2009 pandemic influenza A(H1N1)
- Observational studies published during 2009 and 2010 reporting data on the efficacy, effectiveness, immunological response or adverse effects associated with influenza vaccination of immunocompromised patients to prevent infection from 2009 pandemic influenza A(H1N1)
- Studies which recruited individuals of any age from any setting who are immunocompromised whether due to primary immunodeficiency (genetic defects) or secondary immunodeficiency (such as HIV infection, malignancy, poor nutritional status or use of immunosuppressive drugs)
- No restriction is placed on the influenza vaccination dose, preparation, trade name, schedule or method of administration
- Studies which report data from control or comparator treatments may include no vaccination, placebo vaccination or sham vaccination
- Studies which have recruited immunocompromised patients and compare outcome measures with immunocompetent control study subjects
- Studies which report data on at least one of the following outcome measures: rate of clinically diagnosed influenza or ILI/ITT patients, rate of laboratory confirmed influenza or ITTI patients, immunological response to vaccination, and adverse effects associated with vaccination

- Full text manuscripts of studies which are published in English, French, Spanish, Portuguese, Russian, or Japanese

Exclusion criteria

- Any literature or search hit which does not describe outcome measures obtained from an experimental study, observational study, or systematic review (± meta-analysis)
- Any systematic review (± meta-analysis) which has been superseded by an updated evidence synthesis (such as updated reviews published by the Cochrane Library)
- Studies which report outcome measures associated with vaccination against avian influenza
- Studies which do not report follow-up data of patients within 12 months of intervention
- Studies which have recruited less than 5 subjects to the intervention arm or exposed group
- Studies which have not recruited immunocompromised patients which include those aetiologies described in the protocol
- Studies which compare vaccination with an active comparator and which do not report data from a control group of study subjects
- Studies which compare vaccination only by route of administration or dosing schedules
- Studies which report data from patients with drug induced immunosuppression where less than 80% of the study group are receiving immunosuppressive treatment

A subjective assessment of excluded observational studies published prior to 2009 indexed in MEDLINE will be carried out to confirm the validity of the approach to selection of study design.

One reviewer will execute the search strategy. All identified studies, literature or other documents will be screened by two reviewers for eligibility using a three-stage sifting approach to reviewing the title, abstract and full text. The number of documents identified and screened out will be recorded at each stage (which includes specifying the reason for excluding for studies at the full text stage). Any disagreements will be resolved by discussion or involvement of a third reviewer.

4.0 Assessment of risk of bias and data extraction

In compliance with the PRISMA statement, the risk of bias in individual studies will be assessed at both the study and outcome level.[1] The Cochrane Collaboration tool will be used for assessing the risk of bias in experimental and prospective cohort studies whilst a separate tool developed by

Downs & Black will be used to critique other identified observational studies.[8 9] Systematic reviews which meet the eligibility criteria for inclusion will be assessed for risk of bias using the US Agency for Healthcare Research Quality domain and element-based evaluation instrument.[10] It should be noted that both the Cochrane Collaboration and the PRISMA statement recommend against the use of summary scores for describing an overall risk of bias therefore the result of each assessment will be presented per domain or question.[1 8] Outcome reporting bias will be assessed within each study using the aforementioned quality assessment tools. Data will be extracted from individual studies using a predefined template (see Appendix 2) which will be piloted before the form is locked for a consistent approach to data extraction throughout the systematic review. Data will be collected according to the PICO framework shown above. All quality assessments and data extraction will be conducted independently by two reviewers and any disagreements will be recorded and resolved by discussion or involvement of a third reviewer (CRB, BCM or JVT).

In addition to assessing the risk of bias in non-randomised studies, the Cochrane Collaboration recommends the assessment of confounding due to the potential added risk of selection bias in these studies and importance in contributing towards heterogeneity between studies.[8] Abstracts which meet the protocol eligibility criteria will be subject to data extraction only and not assessment of risk of bias. Reviewers will be required to identify the confounding factors stated in each study, describe the methodology used to measure them, and state how selection bias was controlled through any study design specific features and methods of statistical analysis. In compliance with the Cochrane Collaboration, below is a list of potential confounding factors anticipated to be of importance in the present review (determined based on expert opinion within the review team):

- Aetiology of immunosuppression, including modification of immunosuppressant drug administration
- Demographic characteristics of the study population
- Setting(s) from which study population has been sampled
- Comparative intervention(s) received by control group(s)
- Active influenza vaccination properties, uptake, dosing schedule and route of administration
- Duration of study population follow-up
- Methodology used to assess study outcome measures
- Influenza infection with subtype that active vaccination does not protect against
- Risk factors for exposure to the influenza virus

The risk of bias across studies will be assessed using the approach described by the Grading of Recommendations Assessment, Development and Evaluation (GRADE) working group.[11 12] This process will be conducted independently by two reviewers (CRB and BCM) using publicly available software to produce standardised summary of findings tables for each outcome measure studied (GRADEprofiler v3.2.2, ©GRADE Working Group 2004-2007, available from: http://www.ims.cochrane.org/revman/gradepro [accessed 12 December 2010]). Any disagreements will be recorded and resolved by discussion or involvement of a third reviewer (JVT).

5.0 Data synthesis

Study characteristics and outcome measures will be tabulated to aid narrative synthesis. Statistical analyses will be performed where feasible to produce a meta-analysis of pooled estimates of effect size (including 95% confidence intervals), tests for heterogeneity, and sensitivity analyses. However, further work may be indicated to conduct meta-analyses if there is not sufficient resource within the current project to undertake this. Publication bias will be assessed for each outcome measure studied using funnel plots of effect size versus sample size for each included study (where sufficient data is available).

A narrative approach will be used to synthesise the quality assessments and extracted data according to the following framework described by the Economic and Social Research Council and recommended by the University of York Centre for Reviews and Dissemination:[13]

- Develop a theory of how the intervention works, why and for whom
- Develop a preliminary synthesis of findings of included studies
- Exploring relationships within and between studies
- Assessing the robustness of the synthesis

Sub-group analyses are planned to describe the efficacy and effectiveness of influenza vaccination in patients whose immunodeficiency status is attributable to different aetiologies, and to summarise the available evidence from resource poor settings. Further stratification of sub-group analyses will be presented where sufficient data is available.

6.0 Dissemination

A manuscript will be prepared and submitted for publication in an appropriate peer-reviewed journal. The final version of this manuscript will be submitted to WHO GIP as a key deliverable of the APW in addition to a technical appendix detailing the searched, abstracted and excluded literature content.

An abstract of the systematic review will be submitted for presentation at appropriate health protection or infectious disease conferences during 2011. An oral presentation of this work may also be delivered to WHO GIP.

7.0 Resource implications

The project lead will work closely with the WHO GIP co-ordinator to define the scope and methods of the review. In addition, the WHO GIP co-ordinator will facilitate access to unpublished literature as required and arrange translation of non-English literature. Timescales and key milestones associated with this systematic review will be agreed in discussion by UoN HPRG and WHO GIP.

8.0 References

1. Moher D, Liberati A, Tetzlaff J, Altman DG. Preferred reporting items for systematic reviews and meta-analyses: the PRISMA statement. *BMJ* 2009; **339**: b2535.

2. World Health Organisation. *Standard precautions in health care*. Geneva: World Health Organization; 2007.

3. World Health Organization. *Infection prevention and control of epidemic- and pandemic-prone acute respiratory diseases in health care*. WHO Interim Guidelines: World Health Organization; 2007.

4. World Health Organization. *Infection control recommendations for avian influenza in health-care facilities*. Geneva: World Health Organization; 2008.

5. World Health Organization. *Infection prevention and control during health care for confirmed, probable, or suspected cases of pandemic (H1N1) 2009 virus infection and influenza-like illnesses*. World Health Organization, 2009.

6. World Health Organization. *WHO Guidelines for Pharmacological Management of Pandemic Influenza A(H1N1) 2009 and other Influenza Viruses. Part I Recommendations*. World Health Organization; 2010.

7. Chapter 19 Influenza. In: Salisbury D, Ramsay M, Noakes K (eds). *Immunisation against infectious disease*. Norwich: The Stationary Office; 2006.

8. Higgins J, Green S (eds). *Cochrane Handbook for Systematic Reviews of Interventions*. Version 5.0.2 [updated September 2009]. The Cochrane Collaboration; 2009.

9. Downs SH, Black N. The feasibility of creating a checklist for the assessment of the methodological quality both of randomised and non-randomised studies of health care interventions. *J Epidemiol Community Health* 1998; **52**: 377-384.

10. West S, King V, Carey T, Lohr K, McKoy N, Sutton S, *et al*. *Systems to rate the strength of scientific evidence. Evidence report/technology appraisal number 47*. Agency for Healthcare Research and Quality; 2002.

11. Atkins D, Best D, Briss PA, Eccles M, Falck-Ytter Y, Flottorp S, *et al*. Grading quality of evidence and strength of recommendations. *BMJ* 2004; **328**: 1490.

12. Guyatt GH, Oxman AD, Vist GE, Kunz R, Falck-Ytter Y, Alonso-Coello P, *et al*. GRADE: an emerging consensus on rating quality of evidence and strength of recommendations. *BMJ* 2008; **336**: 924-926.

13. Centre for Reviews and Dissemination. *Systematic reviews. CRD's guidance for undertaking reviews in health care*. York: Centre for Reviews and Dissemination, University of York; 2008.

Appendix 1 – literature search strategy

Search terms

Area	MeSH thesaurus headings	Free text
**Population**	immunosuppression<br>immunocompromised host<br>immunologic deficiency syndromes<br>acquired immunodeficiency syndrome<br><br><br><br><br>phagocyte bactericidal dysfunction<br>HIV<br>tuberculosis<br><br>transplants<br>stem cell transplantation<br><br>neoplasms<br>carcinoma<br>lymphoma<br>leukemia<br>nutrition disorders<br>malnutrition<br>splenic diseases<br>splenectomy<br><br>steroids<br>antineoplastic agents<br>chemotherapy, adjuvant<br>cytotoxicity, immunologic<br>antirheumatic agents<br>immunosuppressive agents	immunosuppress* OR immuno-suppress*<br>immunocompromis* OR immuno-compromis*<br>immun* AND deficien*<br>immunodeficien* OR immuno-deficien*<br>immunoglobulin AND deficien*<br>complement AND deficien*<br>phagocyte AND dysfunction*<br>HIV<br>tuberculosis OR TB<br><br>transplant*<br><br><br>neoplasm* OR cancer<br>carcinoma*<br>lymphoma*<br>leukemi* OR leukaemi*<br>nutrition* AND disorder*<br>malnutrition*<br><br>asplenia<br><br>steroid* OR corticosteroid*<br>antineoplastic AND agent*<br>chemotherap*<br>cytotoxic*
**Intervention**	influenza vaccines<br>viral vaccines<br>vaccines, inactivated<br>vaccines, attenuated<br>adjuvants, immunologic<br>immunization	(influenza OR flu OR season* OR pandemic OR H1N1) AND (vaccin* OR immunis* OR immuniz* OR inoculat*)<br>(inactiv* OR attenu* OR adjuvant*) AND (vaccin* OR immunis* OR immuniz* OR inoculat*)<br>split AND vir*<br>disrupt* AND vir*<br>surface AND antigen* AND inactivat*
**Comparators**	placebos	no AND (vaccin* OR immunis* OR immuniz* OR inoculat*)<br>(placebo OR sham) AND (vaccin* OR immunis* OR immuniz* OR inoculat*)
**Outcomes**	influenza, human<br>influenza A virus	influenza OR flu OR H1N1<br>(influenza-like OR flu-like) OR (influenza

		AND like) OR (flu AND like) AND illness OR ILI
		diagno* AND (influenza OR flu)
		intention-to-treat OR (intention AND to AND treat) OR ITT
		laboratory AND confirm* AND (influenza OR flu)
		(intention-to-treat AND influenza) OR (intention-to-treat AND flu) OR (intention AND to AND treat AND influenza) OR (intention AND to AND treat AND flu) OR ITTI
	hemagglutination inhibition tests<br>immunoglobulin G<br>immunoglobulin A<br>immunoglobulins<br>antibody formation<br>antibodies	((haemagglutin* OR hemagglutin*) AND inhibit* AND anti*) AND HAI<br>(haemagglutin* OR hemagglutin*) OR HA AND (immunoglobulin G OR IgG) AND antibod*<br>(haemagglutin* OR hemagglutin*) OR HA AND (immunoglobulin A OR IgA) AND antibod*
		(adverse AND event*) OR safe* OR (side AND effect*) or (adverse AND effect*) OR harm*

It should be noted that the above MeSH thesaurus headings and free text terms may be amended to maintain compatibility with databases which do not use MeSH or include non-English language studies. Search interfaces with limited functionality (e.g. those which support single line searches only, small number of search terms, etc) may be initially searched using broad influenza-specific terms followed by longer search strings. A list is presented below of example strings which may be used in search interfaces with limited functionality.

- influenza OR flu OR H1N1
- (influenza OR flu OR H1N1) AND (vaccin* OR immunis* OR immuniz* OR inoculat*)
- (((influenza OR flu OR season* OR pandemic OR H1N1 OR inactiv* OR attenu* OR adjuvant*) AND (vaccin* OR immunis* OR immuniz* OR inoculat*) OR (split AND vir*) OR (disrupt* AND vir*) OR (surface AND antigen* AND inactivat*)) AND ((no OR placebo OR sham) AND (vaccin* OR immunis* OR immuniz* OR inoculat*))) AND (influenza OR flu OR H1N1)
- (immunosuppress* OR immuno-suppress* OR immunocompromis* OR immuno-compromis* OR (immun* AND deficien*) OR immunodeficien* OR immuno-deficien* OR (immunoglobulin AND deficien*) OR (complement AND deficien*) OR (phagocyte AND dysfunction*) OR HIV OR

tuberculosis OR TB OR transplant* OR neoplasm* OR cancer OR carcinoma* OR lymphoma* OR leukemi* OR leukaemi* OR (nutrition* AND disorder*) OR malnutrition* OR asplenia OR steroid* OR corticosteroid* OR (antineoplastic AND agent*) OR chemotherap* OR cytotoxic*) AND (((influenza OR flu OR season* OR pandemic OR H1N1 OR inactiv* OR attenu* OR adjuvant*) AND (vaccin* OR immunis* OR immuniz* OR inoculat*) OR (split AND vir*) OR (disrupt* AND vir*) OR (surface AND antigen* AND inactivat*)) OR ((no OR placebo OR sham) AND (vaccin* OR immunis* OR immuniz* OR inoculat*))) AND (influenza OR flu OR H1N1)

All literature searches conducted must be documented (including but not limited to the terms used, sources interrogated and hits identified) such that these may be replicated. When executing the search strategy, the scope of each free text search should include the study title and abstract (where the latter is available). Individual MeSH thesaurus headings and free text terms should be searched for within each group (i.e. population, intervention, comparator, and outcome terms) and subsequently combined using the Boolean operator OR, followed by searches across each group which combine terms in two steps: (1) population AND intervention AND comparator AND outcomes, (2) population AND (intervention OR comparator) AND outcomes. Outcome terms must be combined as ‘flu terms’ OR (‘flu terms’ AND ‘other terms’) where ‘flu terms’ are MeSH thesaurus headings ‘influenza, human’ and ‘influenza A virus’ and the free text term influenza OR flu OR H1N1 and ‘other terms’ refers to all other outcome terms. The two combinations of search terms across the PICO groups must be extracted separately to produce the final list of search hits from each database. All search hits should be imported into reference management software to collate the identified literature and remove duplicates entries prior to conducting the three-stage sifting process. Where search interfaces do not allow the export of search hits to reference management software, search hits may be sifted using alternative electronic or paper-based methods. See below for an example output obtained from executing the search strategy using MEDLINE on 13 January 2011.

Search limits

Limit category	Specified limit
**Languages**	English, Japanese, Russian, French, Spanish, Portuguese
**Publication type**	None
**Date of publication**	No limit is placed on date of publication for experimental studies<br>Observational studies must be published during 2009-2010 to identify literature from the 2009-2010 pandemic period
**Study design**	Experimental studies (seasonal influenza)

	Experimental or observational studies (2009-2010 pandemic period)<br>Observational studies identified with no control group or comparator will be included although these may be associated with a high risk of bias and confounding variables
**Other limits**	Human data

Search sources

Category	Sources
**Healthcare databases**	MEDLINE<br>EMBASE<br>CINAHL<br>Cochrane Library (CENTRAL)<br>PubMed (includes MEDLINE)<br>WHO Regional Indexes including the African Index Medicus, Index Medicus for the Eastern Mediterranean Region, Index Medicus for South-East Asia Region, Latin American and Caribbean Health Science Information, and the Western Pacific Region Index Medicus<br>J-STAGE (includes Japanese literature)<br>BDSP (includes French literature)<br>Index-F (includes Spanish literature)<br>eLIBRARY (includes Russian literature)
**Evidence based reviews**	Bandolier<br>Cochrane Library (CDSR, DARE, NHS HTA database)
**Guidelines**	NHS Evidence (NHS Clinical Knowledge Summaries and the National Library of Guidelines)
**Grey literature**	Web of Science<br>NHS Evidence (drug information, evidence summaries, grey literature, health technology assessments, primary research, and systematic reviews)<br>OpenSIGLE (system for information on grey literature in Europe)<br>Influenza vaccine manufacturers (GlaxoSmithKline, Novartis, Sanofi Pasteur MSD, Abbott, CSL Limited, Medimmune, Crucell, Baxter)<br>EVM (European Vaccine Manufacturers, Brussels), IFPMA (International Federation of Pharmaceutical Manufacturers Associations, Geneva/Zurich)<br>Consultation with domain expert – Bram Palache (Abbott)
**Hand searching of relevant journals**	*Vaccine*
**Reference tracking**	Reference lists of all studies selected for inclusion will be searched to identify further relevant studies
**Citation tracking**	Web of Science (Science Citation Index)<br>Google Scholar
**Internet searching**	www.google.com<br>www.dh.gov.uk<br>www.hpa.org.uk<br>www.who.int<br>www.cdc.gov<br>www.flu.gov

Example search output – MEDLINE (conducted 13 January 2011)

No.	Search term	Hits
1	IMMUNOSUPPRESSION/	25561
2	IMMUNOCOMPROMISED HOST/	11634
3	IMMUNOLOGIC DEFICIENCY SYNDROMES/	11729
4	ACQUIRED IMMUNODEFICIENCY SYNDROME/	68860
5	PHAGOCYTE BACTERICIDAL DYSFUNCTION/	589
6	HIV/	13550
7	TUBERCULOSIS/	43649
8	TRANSPLANTS/	1510
9	STEM CELL TRANSPLANTATION/	11727
10	NEOPLASMS/	222137
11	CARCINOMA/	55690
12	LYMPHOMA/	40523
13	LEUKEMIA/	44518
14	NUTRITION DISORDERS/	16833
15	MALNUTRITION/	3991
16	SPLENIC DISEASES/	5606
17	SPLENECTOMY/	17096
18	STEROIDS/	25233
19	ANTINEOPLASTIC AGENTS/	146151
20	CHEMOTHERAPY, ADJUVANT/	23207
21	CYTOTOXICITY, IMMUNOLOGIC/	29616
22	ANTIRHEUMATIC AGENTS/	10508
23	IMMUNOSUPPRESSIVE AGENTS/	61805
24	(immunosuppress* OR immuno-suppress*).ti,ab	85669
25	(immunocompromis* OR immuno-compromis*).ti,ab	16556
26	(immun* AND deficien*).ti,ab	53851
27	(immunodeficien* OR immuno-deficien*).ti,ab	94212
28	(immunoglobulin AND deficien*).ti,ab	4305
29	(complement AND deficien*).ti,ab	6110
30	(phagocyte AND dysfunction*).ti,ab	146
31	HIV.ti,ab	179116
32	(tuberculosis OR TB).ti,ab	122306
33	transplant*.ti,ab	275704
34	(neoplasm* OR cancer).ti,ab	832493
35	carcinoma*.ti,ab	388780
36	lymphoma*.ti,ab	106492
37	(leukemi* OR leukaemi*).ti,ab	177539
38	(nutrition* AND disorder*).ti,ab	6170
39	malnutrition*.ti,ab	22259
40	asplenia.ti,ab	689
41	(steroid* OR corticosteroid*).ti,ab	207968
42	(antineoplastic AND agent*).ti,ab	5437
43	chemotherap*.ti,ab	212862
44	cytotoxic*.ti,ab	155554
45	1 OR 2 OR 3 OR 4 OR 5 OR 6 OR 7 OR 8 OR 9 OR 10 OR 11 OR 12 OR 13 OR 14 OR 15 OR 16 OR 17 OR 18 OR 19 OR 20 OR 21 OR 22 OR 23 OR 24 OR	2561850

	25 OR 26 OR 27 OR 28 OR 29 OR 30 OR 31 OR 32 OR 33 OR 34 OR 35 OR 36 OR 37 OR 38 OR 39 OR 40 OR 41 OR 42 OR 43 OR 44	
**46**	INFLUENZA VACCINES/	11682
**47**	VIRAL VACCINES/	17421
**48**	VACCINES, INACTIVATED/	3060
**49**	VACCINES, ATTENUATED/	7670
**50**	ADJUVANTS, IMMUNOLOGIC/	27422
**51**	IMMUNIZATION/	38677
**52**	((influenza OR flu OR season* OR pandemic OR H1N1) AND (vaccin* OR immunis* OR immuniz* OR inoculat*)).ti,ab	18880
**53**	((vaccin* OR immunis* OR immuniz* OR inoculat*) AND (inactiv* OR attenu* OR adjuvant*)).ti,ab	36649
**54**	(split AND vir*).ti,ab	1251
**55**	(disrupt* AND vir*).ti,ab	9617
**56**	(surface AND antigen* AND inactivat*).ti,ab	1011
**57**	46 OR 47 OR 48 OR 49 OR 50 OR 51 OR 52 OR 53 OR 54 OR 55 OR 56	133351
**58**	PLACEBOS/	28797
**59**	(no AND (vaccin* OR immunis* OR immuniz* OR inoculat*)).ti,ab	51906
**60**	((placebo OR sham) AND (vaccin* OR immunis* OR immuniz* OR inoculat*)).ti,ab	3682
**61**	58 OR 59 OR 60	82640
**62**	INFLUENZA, HUMAN/	24968
**63**	INFLUENZA A VIRUS/	13855
**64**	HEMAGGLUTINATION INHIBITION TESTS/	11784
**65**	IMMUNOGLOBULIN G/	97983
**66**	IMMUNOGLOBULIN A/	27424
**67**	IMMUNOGLOBULINS/	36984
**68**	ANTIBODY FORMATION/	55569
**69**	ANTIBODIES/	78321
**70**	(influenza OR flu OR H1N1).ti,ab	53816
**71**	((influenza-like OR flu-like) OR (influenza AND like) OR (flu AND like) AND illness OR ILI).ti,ab	1633
**72**	(diagno* AND (influenza OR flu)).ti,ab	4154
**73**	(intention-to-treat OR (intention AND to AND treat) OR ITT).ti,ab	9158
**74**	(laboratory AND confirm* AND (influenza OR flu)).ti,ab	667
**75**	((intention-to-treat AND influenza) OR (intention-to-treat AND flu) OR (intention AND to AND treat AND influenza) OR (intention AND to AND treat AND flu) OR ITTI).ti,ab	43
**76**	(((haemagglutin* OR hemagglutin*) AND inhibit* AND anti*) AND HAI).ti,ab	354
**77**	((haemagglutin* OR hemagglutin*) OR HA AND (immunoglobulin G OR IgG) AND antibod*).ti,ab	1831
**78**	((haemagglutin* OR hemagglutin*) OR HA AND (immunoglobulin A OR IgA) AND antibod*).ti,ab	1257
**79**	((adverse AND event*) OR safe* OR (side AND effect*) OR (adverse AND effect*) OR harm*).ti,ab	700294
**80**	62 OR 63 OR 70	58457
**81**	64 OR 65 OR 66 OR 67 OR 68 OR 69 OR 71 OR 72 OR 73 OR 74 OR 75 OR 76 OR 77 OR 78 OR 79	965600

**82**	80 OR (80 AND 81)	58457
**83**	45 AND 57 AND 61 AND 82	509
**84**	45 AND (57 OR 61) AND 82	2547
**85**	45 AND (57 OR 61) AND 82 [Limit to: Publication Year 2009-2010]	407
**86**	83 [Limit to: Humans and (Languages English or French or Japanese or Portuguese or Russian or Spanish)]	379
**87**	85 [Limit to: Humans and (Languages English or French or Japanese or Portuguese or Russian or Spanish) and Publication Year 2009-2010]	289

Appendix 2 – data extraction form

Reviewers will be provided with a template Microsoft® Excel workbook to complete and return all assessments of risk of bias and extracted data from individual studies. The table below provides version control of the workbook used throughout this systematic review.

Data extraction form	First date in use	Reason for modification
15 December 2010, v1	15 December 2010	N/A (first version, unpiloted)
2 February 2011, v2	2 February 2011	Amendments made following feedback after piloting process
